# HCN2 Rescues brain defects by enforcing endogenous voltage pre-patterns

**DOI:** 10.1038/s41467-018-03334-5

**Published:** 2018-03-08

**Authors:** Vaibhav P. Pai, Alexis Pietak, Valerie Willocq, Bin Ye, Nian-Qing Shi, Michael Levin

**Affiliations:** 1 Allen Discovery Center at Tufts University, Medford, MA 02155 USA; 2 Veridian Biotechnology Limited, Biotech Center 2, Hong Kong, China; 3 Department of Developmental, Molecular, and Chemical Biology, Tufts University, Boston, MA 02111 USA

## Abstract

Endogenous bioelectrical signaling coordinates cell behaviors toward correct anatomical outcomes. Lack of a model explaining spatialized dynamics of bioelectric states has hindered the understanding of the etiology of some birth defects and the development of predictive interventions. Nicotine, a known neuroteratogen, induces serious defects in brain patterning and learning. Our bio-realistic computational model explains nicotine’s effects via the disruption of endogenous bioelectrical gradients and predicts that exogenous HCN2 ion channels would restore the endogenous bioelectric prepatterns necessary for brain patterning. Voltage mapping in vivo confirms these predictions, and exogenous expression of the HCN2 ion channel rescues nicotine-exposed embryos, resulting in normal brain morphology and molecular marker expression, with near-normal learning capacity. By combining molecular embryology, electrophysiology, and computational modeling, we delineate a biophysical mechanism of developmental brain damage and its functional rescue.

## Abstract (web summary)

The authors have previously shown that membrane voltage can influence embryonic patterning during development. Here, the authors computationally model how nicotine disrupts *Xenopus* embryogenesis by perturbing voltage gradients, and rescue nicotine-inducted defects with HCN2 channel expression.

## Introduction

Improper neural patterning during development leads to highly debilitating disorders, such as open neural tube defects [spina bifida, and anencephaly (small brain)]^1^, brain malformations^2^, and susceptibility to autism and degenerative disorders like Parkinson’s and Alzheimer’s diseases^3–5^. There are not currently any clinically approved interventions able to rescue such brain patterning disorders. Finding regenerative and repair strategies for brain patterning and function is a critical unmet need in developmental and regenerative medicine.

In addition to well-known growth factors and chemical pathways, endogenous bioelectrical signals (spatiotemporal distributions of resting membrane voltage—*V*_mem_—across tissues) have been shown to regulate aspects of large-scale patterning during embryonic development^6–13^. Endogenous bioelectric signals (produced by the function of ion channels and pumps) have long been known to have roles in cell migration, wound healing, and the direction of growth and form in vivo^14,15^. Bioelectric signals are implicated in vertebrate appendage regeneration and development^16–18^, craniofacial morphogenesis^19,20^, left-right patterning^21,22^, cancer^23^, heart and muscle patterning^24,25^, planarian head regeneration^26^, and eye and brain patterning^27,28^. Here, we explore bioelectric signaling as both a target of teratogens that induce brain defects and as a target for therapeutic strategies to rescue such malformations.

Recent studies from our laboratory and other groups (reviewed in Ref. ^15^) identified molecular mechanisms by which bioelectrical signaling participates in development (including transduction machinery and transcriptional targets). However, the state-of-the-art for in vivo data in this exciting field has been “arrow diagrams” that identify the cellular-level bioelectric pathways of gene products and signals required for development or regeneration. The field still largely lacks knowledge of the quantitative dynamics that are sufficient to produce the spatial patterning across tissues or organs. Widely recognized as the next key step, but still missing, are biorealistic multi-scale models that integrate cell-level electrophysiology and molecular biology with large-scale patterning information (molecular, anatomical, and bioelectric). Such a synthesis is required in order to explain, in a rigorous manner, how bioelectric events originating with ion channel proteins scale up to regulate emergent organ patterning in health and disease. Here, we present and analyze the first example of such a model in the context of teratogenesis of the *Xenopus* brain and use it to identify a successful repair strategy.

Nicotine is a well-known neuroteratogen: embryonic exposure to nicotine leads to severe brain morphological defects as well as significant postnatal deficits in cognitive functions^29–32^. The majority of these effects of nicotine occur through its action on nicotinic acetyl choline (nAChR) receptors, but it is not known how this bioelectric change at the single-cell level alters whole organ morphogenesis of the brain. Here, we establish an amphibian model for nicotine teratogenesis, and exploit the *Xenopus* embryo as proof-of-principle of how computational models of physiological regulatory events can help identify mechanisms of developmental defects and drive the development of therapeutic strategies targeting endogenous bioelectricity.

Hyperpolarization-activated cyclic nucleotide-gated (HCN) channels are a group of voltage-gated ion channels in which the threshold voltage is modulated by the metabolic state of the cell (levels of cyclic nucleotides, like cAMP)^33^, thus allowing them to regulate cellular voltage in a context-specific manner. They open at hyperpolarized (negative) *V*_mem_, giving rise to currents that are a mix of sodium and potassium fluxes. Among the four HCN isoforms (HCN1–4), HCN2 channels show an especially high sensitivity to cAMP. These channels are primarily present in the adult nervous system and heart, but have also been detected in human and mouse embryonic cells^34–37^. To the best of our knowledge, these channels have not been studied in the context of embryonic brain development or as targets for developmental therapeutics.

Here we present molecular physiology data and computational analysis explaining neuroteratogen nicotine-induced embryonic brain mispatterning. We report the first biorealistic computational model of *Xenopus* embryos, which predicts that nicotine disrupts the endogenous bioelectric patterns critical for brain patterning. This mispatterning mechanism is validated using voltage reporter dyes, and we model the consequences of possible methods for manipulating bioelectric gradients in vivo. Our model predicts that rescue would be effected by restoring this embryonic bioelectrical pattern via a reagent with context-specific effects on resting potential: the HCN2 channel. Remarkably, we find that misexpressing HCN2 channels in nicotine-exposed embryos rescues the endogenous spatial voltage gradient in the nascent neural tube and leads to a correction of the expression patterns of key brain transcription factors and a near complete rescue of brain morphology defects and cognitive learning abilities. Our integration of molecular developmental biology and simulation provides insight into the mechanisms of brain development and teratogenesis, and illustrates proof of principle of using computational modeling to understand and manipulate endogenous bioelectrical signaling in the context of regenerative medicine.

## Results

### Embryonic nicotine exposure induces brain morphology defects

**Fig. 1:**
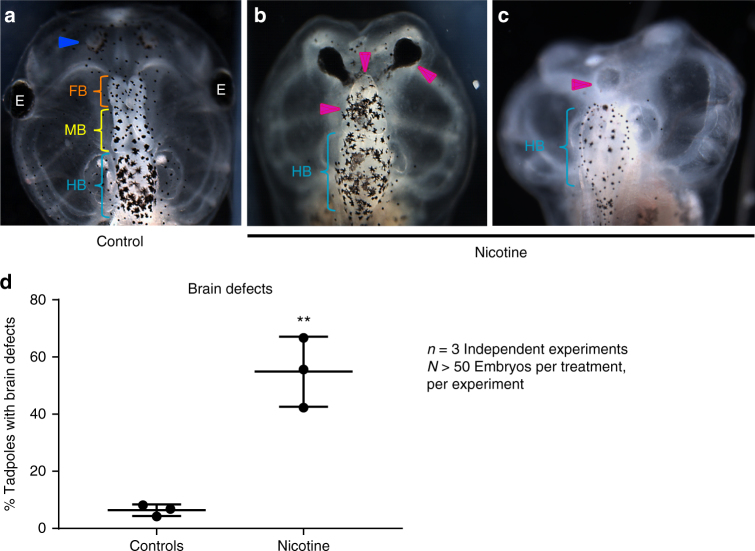
Nicotine induces brain morphology defects in *Xenopus* embryos. Representative images of stage 45 tadpoles: **a** control tadpole showing nostrils (blue arrowhead), forebrain (FB) indicated by the orange bracket, midbrain (MB) indicated by the yellow bracket, and hind brain (HB) indicated by the cyan bracket, **b** and **c** tadpoles from embryos exposed to nicotine (0.1 mg/mL – stage 10–35) showing severe brain morphology defects as indicated by magenta arrowheads. Cyan brackets indicate presence of hindbrain (HB). **d** Quantification of stage 45 tadpoles for major brain morphology phenotypes in absence or presence of nicotine exposure (0.1 mg/mL – stage 10–35). A significantly high incidence of malformed brain was observed in embryos exposed to nicotine in comparison to controls. Three independent experiments (*n* = 3) were conducted with *N* > 50 embryos per treatment group for each of those experiments collected from multiple animals across independent clutches. Data were analyzed with t-test and graphed as mean ± SD; **p < 0.01

Nicotine is a well-known developmental neuroteratogen in humans^29^, causing morphological defects, decreased cognitive functions, and deficits in learning and memory. We first sought to establish a frog model of nicotine teratogenesis by exposing *Xenopus* embryos to nicotine (0.1 mg/mL). Nicotine exposure was targeted to stages 10–35, when neural tissue induction and patterning occurs. Untreated embryos served as controls, as neither ethanol nor dimethyl sulfoxide (DMSO) was needed to formulate the stock solution of nicotine used in our assays. Brain morphology of the embryos was assessed after they had developed to stage 45 (Fig. 1). Control tadpoles had correctly-patterned brain tissue^5^, with well-formed nostrils, olfactory bulbs/forebrain, midbrain, and hindbrain (Fig. 1a, d). Nicotine exposure caused a significantly high incidence of major brain morphology defects (~55%) in comparison to controls (~6%). The most striking phenotypes were the absence of nostrils, absence of forebrain, absence of both forebrain and midbrain, and occasionally misformed hindbrain (Fig. 1b, c) or truncated spinal cord. The hindbrain was the least affected of the brain regions. Eye development requires proper neural induction and development^38,39^. Tadpoles displayed abnormalies including missing eyes, incompletely formed eyes, fusion of eye to the brain, and pigmented optic nerves (Fig. 1b, c; see also ref. ^27^). We conclude that nicotine is a powerful neuroteratogen in *Xenopus*.

### Model of *V*_mem_ gradients patterning the embryonic brain

Our previous work demonstrated that specific *V*_mem_ distributions (pre-patterns) within the developing neural tube and surrounding regions are critical for proper patterning of the developing brain^28^, while other studies have characterized specific ion channels in developing of neuron populations^40^. No existing spatial model quantitatively synthesizes the functional and electrophysiological data to explain the control of patterning by endogenous ion channel activity; such a model is needed to understand the biophysics of organ development and to uncover mechanisms of action of ion channel-targeting teratogens. As bioelectric circuits can have highly non-obvious behavior, we used a computational platform, the BioElectric Tissue Simulation Engine (BETSE^41^), to characterize the dynamics of voltage gradients under the relevant conditions in vivo, and to develop a predictive physiological model (see Supplementary Note). BETSE is a software simulator specially designed to study bioelectricity from a “first principles” perspective, which focuses on ion concentrations, fluxes, and transport using realistic parameters derived from molecular physiology studies of cells to gain insight into spatialized dynamics of relevance to pattern formation^41^.

**Fig. 2:**
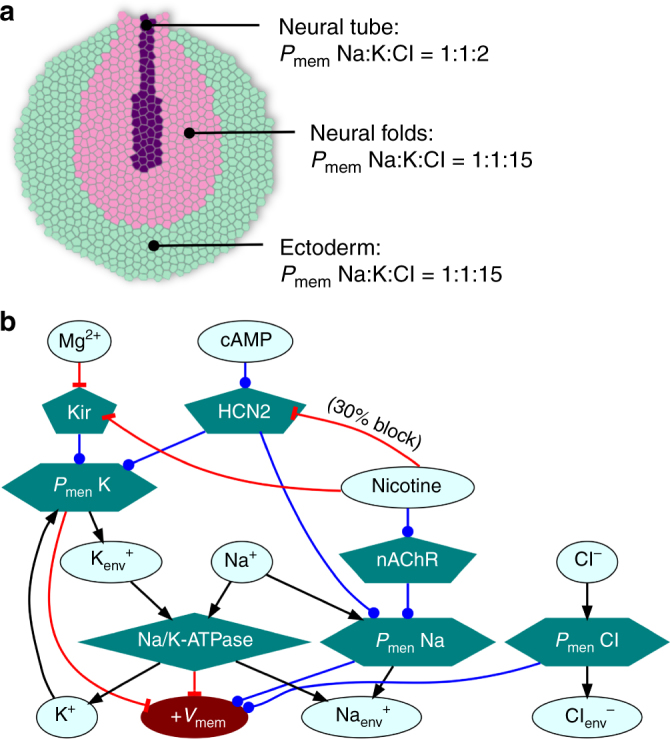
BETSE model of endogenous resting potential gradients that instructively pattern embryonic neural tissue. **a** The BETSE model of neurula stage *Xenopus* embryo featured three different cell groups (referred to as tissue profiles) as indicated by color and labels. The neural tube profile was made to have 5× higher leak membrane permeability to K^+^ ions and 15× lower membrane permeability to Cl^−^ ions, compared to the other two profiles, leading to hyperpolarized resting *V*_mem_ in neural tube cells as has been experimentally observed^28, 48^. The model is assumed to be the outer layer of cells which face 0.1 × MMR. See supplemental document for detailed description of model. **b** The regulatory network describing the main components of the model. All cells of all simulations expressed equal levels of Na/K-ATPase ion pumps, inward-rectifying K^+^ channels (Kir), and nAChR cation channels. Some simulations included HCN2 channels equally expressed on all cells, and/or nicotine introduced via environmental exposure

BETSE software was used to construct models of *V*_mem_ patterns in the early *Xenopus* neurula and to examine the influence of various interventions (Supplementary Note). Initial (i.e., simulation time zero) ion and substance concentrations in the cytosol, intercellular space, and global environmental media are summarized in Table 1. BETSE modeled an exterior sheet of cells representing an anterior view of a stage 15 *Xenopus* neurula, where cells were assumed to be a 10-μm-thick layer facing 0.1 × MMR (Fig. 2a). Voltage-sensitive gap junctions (GJs) were employed in all models, using the kinetic model of voltage sensitivity fit to amphibian gap junctions^42^.

**Table 1:** Initial simulation concentrations in the intracellular space (cytosol), extracellular space (intercellular regions), and global environmental space surrounding cells (0.1 × MMR)

Ion	Intracellular (mM)	Extracellular (mM)	0.1 × MMR (mM)
Na^+^	21.3	101.8	10.0
K^+^	91.0	3.7	0.5
Cl^−^	40.2	37.6	9.0
Ca^2+^	50e-6	1.5	0.1
Anionic protein	47.0	10.0	0.0
ATP	2.5	0.0	0.0
ADP	0.1	0.0	0.0
P_i_	0.1	0.0	0.0
H^+^	4.0e-5	4.0e-5	4.0e-5
HCO_3_^−^	10.0	10.0	1.0
Charge balancing anions	15.1	47.9	1.5

**Fig. 3:**
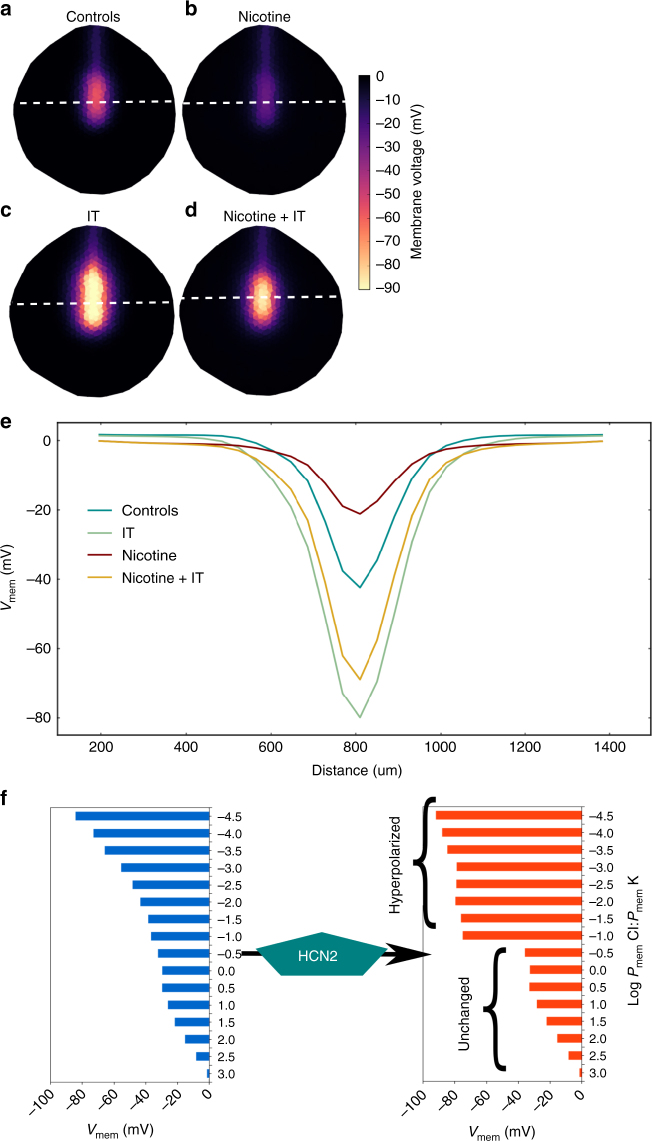
BETSE model predicts that nicotine suppresses neural tube hyperpolarization and HCN2 recovers neural tube hyperpolarization in presence of nicotine. **a**–**e** BETSE model of a stage 15 *Xenopus* embryo where cells are assumed to be facing 0.1 × MMR. **a** Control model exhibits a characteristic *V*_mem_ pattern featuring relative hyperpolarization in the neural tube area and depolarization everywhere else. This *V*_mem_ pattern is actually seen in the *Xenopus* embryos with voltage reporter dyes^28^. **b** Nicotine treatment of the control model is predicted to preferentially depolarize the neural tube area to suppress the *V*_mem_ gradient. **c** Model with ion translocator (IT) expression in all cells, acting in a context-specific manner showing strongly enhanced neural tube *V*_mem_ pattern. **d** Adding the ion translocator (IT) to the nicotine treated model shows the *V*_mem_ pattern significantly retained in comparison to the only nicotine treated model of **b**. **e** Line graph showing the information retrieved along the white dotted lines depicted in (**a**–**d**), which allows for easy comparison between models. **f** The effect of the HCN2 channel on the *V*_mem_ of a series of cells with different membrane leak permeabilities (*P*_mem_). The ability of homogeneously expressed HCN2 channels to act in a context specific manner is predicted to stem from the channels’s opening at hyperpolarized *V*_mem_ with a hyperpolarizing effect upon opening. This selectively hyperpolarizes cells while leaving relatively more depolarized cell’s *V*_mem_ unchanged. This further emphasizes that HCN2 channels amplify the endogenous *V*_mem_ pattern of the neural tube and maintain the *V*_mem_ pattern against nicotine depolarization exactly like IT in (**c**, **d** and **e**)

A depiction of the complete bioelectric regulatory network underlying all BETSE models reported herein (including the optional elements: nicotine, IT, and HCN2 channels) is shown in Fig. 2b (Supplementary Note). The control model represents the fundamental (endogenous baseline) physiological situation, without nicotine treatment or IT misexpression. The control model considered three different spatial regions of cells, roughly demarcating the ectoderm, neural folds, and neural tube areas of the embryo from an anterior view of the outer surface (Fig. 2a). Variations in the base level of membrane permeability to K^+^ and Cl^−^(Fig. 2a) simulate the presence of leak channels, creating the observed endogenous relative hyperpolarization in the neural tube compared to the neural folds and ectoderm. This results in open Kir channels in the hyperpolarized neural tube region leading to a *V*_mem_ pattern of approximately −50 mV in the neural tube region, while the lateral neural folds and ectoderm remain at ~−5 mV (Fig. 3a, e). This hypothesis of generation of the endogenous *V*_mem_ pattern in the *Xenopus* neural tube is consistent with observed *V*_mem_ patterns in *Xenopus* embryos and the effect of dominant-negative Kir channels on them^27,28^.

### Nicotine targets *V*_mem_ prepattern to disrupt brain patterning

**Fig. 4:**
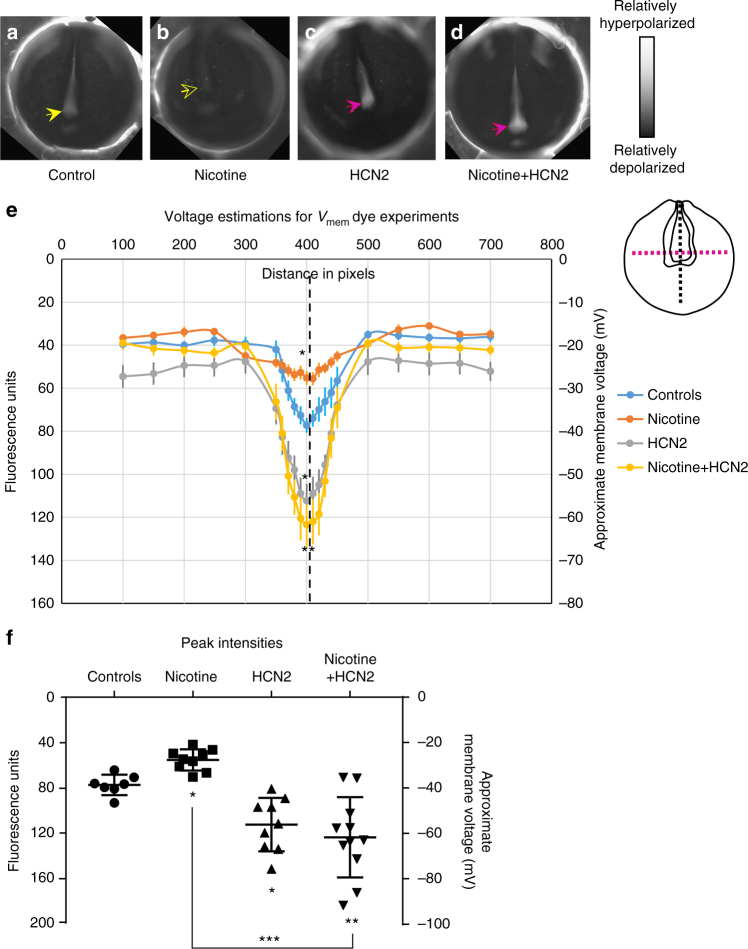
Validation of the model prediction of nicotine effects and HCN2-induced recovery of membrane voltage prepatterns. Representative CC2-DMPE:DiBAC_4_(3) images of stage ~15 *Xenopus* embryos: **a** untreated controls, **b** nicotine-exposed (0.1 mg/mL – stage 10–35), **c** *Hcn2-WT* mRNA microinjected (0.75 ng/injection) in both blastomeres at 2-cell stage, and **d** nicotine-exposed (0.1 mg/mL – stage 10–35) and *Hcn2-WT* mRNA microinjected (0.75 ng/injection) in both blastomeres at 2-cell stage. Control embryos show the characteristic hyperpolarization (solid yellow arrow) as previously reported^28^. Nicotine-treated embryos show reduced signal (depolarized) within the neural tube (hollow yellow arrow). *Hcn2-WT* mRNA microinjection show enhanced signal (hyperpolarized) within the neural tube (magenta arrows), in presence or absence of nicotine exposure. **e** Quantification of CC2-DMPE:DiBAC_4_(3) images of stage ~15 *Xenopus* embryos along the red dotted line as indicated in the inset illustration, along with electrophysiology based membrane voltage approximations (as previously reported in refs.^27,28^). The fluorescence intensity/membrane voltage pattern within the neural tube (indicated by the black dotted line in the inset illustration and corresponding lack dotted line in the graph) is significantly reduced (depolarization) in nicotine-exposed embryos in comparison to controls. *Hcn2-WT* mRNA microinjection significantly enhances the fluorescence intensity/membrane voltage patterns within the neural tube in comparison to controls. Nicotine-exposed embryos that are microinjected with *Hcn2-WT* mRNA maintain a significantly enhanced fluorescence intensity/membrane voltage pattern within the neural tube in comparison to only nicotine-treated embryos. *N* = 10 embryos for each treatment group at each of the indicated spatial distance in pixels were collected from multiple animals across independent clutches. Data is plotted as mean ± S.E.M. Data at black line (400 Pixels) was analyzed using one way ANOVA, *p < 0.05, **p < 0.01. (**f**) Quantification of peak fluorescence intensity and electrophysiology based membrane voltage approximations (as previously reported in ref. ^27,28^) from voltage reporter dye images (CC2-DMPE:DiBAC_4_(3)) of ~stage 15 *Xenopus* embryos within the neural tube at the intersection of the red and black dotted lines in the inset illustration in (**a**). Nicotine exposure significantly reduced (depolarizes) the neural tube peak intensity/membrane voltage in comparison to controls. *Hcn2-WT* mRNA microinjection, both in presence or absence of nicotine, significantly enhances (hyperpolarizes) the neural tube peak intensity/membrane voltage in comparison to controls. *N* = 10 embryos for each treatment group at each of the indicated spatial distance in pixels were collected from multiple animals across independent clutches. Data is plotted as mean ± SD and was analyzed using one way ANOVA, *p < 0.05, **p < 0.01, ***p < 0.001

Simulation of nicotine exposure was built upon the control model by incorporation of nicotine-sensitive ionotropic nAChR receptors, expressed homogenously throughout the embryo^43^ (Supplementary Note). While nicotine is a well-known agonist of the nicotinic acetylcholine receptor (nAChR)^44^, it can also directly block a variety of K^+^ ion channels^45^, including the HCN channels^46^. Nicotine block of HCN channels is only partial (<39%). All this information was included in the models using a standard Hill function to describe the influence of nicotine on the channel state as a function of its concentration (Supplementary Note). Nicotine exposure simulated 0.62 mM diffusion of nicotine from the environment, which the model predicted would depolarize the *V*_mem_ by increasing Na^+^ membrane permeability (Fig. 3b, e). Treating the model with nicotine predicted to diminish the endogenous neural tube hyperpolarization pattern in comparison to controls (Fig. 3a, b, and e), thus reducing the spatial contrast of *V*_mem_ between the hyperpolarized neural tube and the depolarized neural folds and ectoderm (Fig. 3e). We tested the BETSE model predictions about nicotine effect on *V*_mem_ patterns in stage 15 embryos, using a combination of in vivo voltage reporter dye (CC2-DMPE:DiBAC_4_(3)) imaging^47^ and whole cell *V*_mem_ recordings^27,28^. Whole-cell electrophysiological recordings of *V*_mem_ from neural plate cells and flanking ectodermal cells provided calibration points for the voltage reporter dye images; the fluorescence intensities were analyzed against these calibration points to approximate membrane voltages at different points within the developing embryos (Fig. 4 and Supplementary Figure 1). Nicotine-treated embryos showed significant reduction in endogenous neural *V*_mem_ signal (depolarization by ~15 mV) in comparison to controls (Figs. 4a, b, e, f, and Supplementary Figure 1). This observation matches the BETSE model predictions of nicotine effect on *V*_mem_ both in direction (depolarization) and magnitude (~15 mV). Our model explains why nicotine induces brain defects: it correctly predicts the disruptive effects of this teratogen on the endogenous bioelectric prepattern required for brain development.

### BETSE model identifies HCN2 channel as a repair reagent

Simulations of *V*_mem_ manipulation using an ion translocator (IT) involved the use of the control model with IT equally expressed in all cells. Simulations indicated that a homogenously expressed IT that opened preferentially in the hyperpolarized neural tube region, while remaining closed in the two lateral regions of the model, would lead to accentuation of neural tube *V*_mem_ pattern to ~−80 mV, while keeping remaining two regions of the model at ~−5 mV (Fig. 3c, e)—the spatial prepattern observed in wild-type embryos. The spatial patterning is crucial, as both depolarization of the middle region, or hyperpolarization of the lateral regions, both cause brain defects—it is the precise differential bioelectric state that induces the required balance of proliferation, apoptosis, and gene expression to build a normal brain^28,48^. Our model predicted that misexpression of an IT that amplified endogenous *V*_mem_ patterns (hyperpolarization) in the neural tube without affecting the *V*_mem_ patterns in the surrounding ectoderm would lead to maintenance of the endogenous neural tube *V*_mem_ pattern even in nicotine-exposed model embryos (Fig. 3c vs. d, e).

BETSE predicted HCN2 channels as an ideal reagent to meet this requirement. Its *V*_mem_ pattern amplification arises from the fundamental dynamics of the HCN2 channel in 0.1 × MMR (which is a low ionic environment). HCN2 channels (mostly studied in adult mammalian cardiac and neural tissues), in conjugation with voltage-gated Na^+^ and K^+^ channels, create self-oscillating *V*_mem_ signals^33^. The frequency of these *V*_mem_ oscillations is regulated by cAMP, which shifts the opening threshold of HCN2 channels to more positive *V*_mem_ values and couples HCN2-mediated *V*_mem_ oscillation frequency to cAMP modulators and cell metabolism^33,49^. Under *Xenopus* ionic parameters and external media, HCN2 channels hyperpolarize (Table 1 and Fig. 3f), which is also consistent with predictions using the GHK equation. HCN channels are primarily permeable to Na^+^ and K^+^ in a Na^+^:K^+^ ratio of ~0.2–0.33 (ref. ^33^). In this first model of the role of HCN2 channels in *Xenopus* development, the voltage and cAMP sensitivity of HCN2 channels were accounted for using kinetic models of HCN2 voltage sensitivity^50^ in combination with the assumption that the presence of cAMP shifts the *V*_1/2_ opening threshold of the HCN2 channel by + 20 mV (ref. ^49^) from its baseline value of *V*_1/2_ = 99.0 in the absence of cAMP^50^. HCN2 channel models used an Na^+^:K^+^ permeability ratio of 0.2 (ref. ^33^).

Our modeling predicted that HCN2 would be able to amplify the endogenous *V*_mem_ gradient patterns in *Xenopus* neurula (Fig. 3f). HCN2 channels are predicted to open at hyperpolarized *V*_mem_, generating more hyperpolarization with opening, thereby serving as a *V*_mem_-controlled switch of *V*_mem_ state, capable of amplifying small *V*_mem_ gradients, similar to IT effect (Fig. 3c, e, f). Thus, HCN2 channel transitions the *V*_mem_ of sufficiently electronegative cells to very hyperpolarized state, while leaving the *V*_mem_ of other relatively depolarized regions unchanged (Fig. 3c, e, f). Simulations indicated that homogenously expressed HCN2 channels open preferentially in the neural tube region, while remaining closed in the other two regions of the model. This leads to accentuation of neural tube *V*_mem_ pattern ~−80 mV, while keeping remaining two regions of the model at ~−5 mV, similar to IT (Fig. 3c, e, f). Importantly, our model predicts that HCN2 expression leads to maintenance of neural tube *V*_mem_ pattern even in nicotine-exposed model embryos, similar to the action of IT (Fig. 3c vs. d, e, f). This result suggests that HCN2 channels should amplify the intrinsic *V*_mem_ pattern of the neural tube, leading to a maintenance of *V*_mem_ pattern in spite of the depolarizing influence of nicotine. Thus, our computational model begins with a quantitative physiological explanation of the brain defects induced by nicotine and identifies an intervention strategy, predicting repair by the HCN2 channel.

### HCN2 corrects *V*_mem_ pattern in nicotine-exposed embryos

To test the predictions made by the BETSE model about nicotine’s and HCN2’s effects on membrane voltage prepatterns, we evaluated embryos between stage 15–17 using a combination of in vivo imaging with voltage reporter dye pair CC2-DMPE:DiBAC_4_(3) (ref. ^47^) and whole cell *V*_mem_ recordings (Fig. 4; refs. ^27,28,]^). Whole-cell electrophysiological recordings of *V*_mem_ from neural plate cells and flanking ectodermal cells were used as calibration points for the voltage reporter dye images, and the fluorescence intensities were analyzed against these calibration points to approximate membrane voltages at different points within the developing embryos (Fig. 4 and Supplementary Figure 1). Uninjected and untreated embryos (controls), embryos exposed to nicotine (stage 10 onward), embryos microinjected with *Hcn2-WT (wild type)* mRNA (both blastomeres at two-cell stage for an overall uniform expression) and embryos exposed to nicotine that are also injected with *Hcn2-WT* mRNA were analyzed.

Nicotine-treated embryos showed significant reduction in endogenous neural *V*_mem_ signal (depolarization by ~15 mV) in comparison to controls (Fig. 4a, b, e, f, and Supplementary Figure 1). This observation matches the BETSE model predictions of nicotine effect on *V*_mem_ both in direction (depolarization) and extent (~15 mV). Consistent with the BETSE model, voltage imaging in *Hcn2-WT*-injected embryos revealed a significantly enhanced endogenous neural signal (hyperpolarization by ~15 mV) in comparison to controls (Fig. 4a, c, e, f, and Supplementary Figure 1). However, the extent of enhanced hyperpolarization predicted by BETSE model for IT and HCN2 was higher (~30 mV) than actually observed (~15 mV) (Figs. 3e and 4). Crucially, nicotine-exposed embryos that were injected with *Hcn2-WT* channel mRNA showed remarkably enhanced (hyperpolarized better than controls by ~20 mV) endogenous neural *V*_mem_ signals in comparison to only nicotine treated embryos (hyperpolarized by ~30 mV) (Fig. 4a, b, d, e, f, and Supplementary Figure 1). This observation largely fits BETSE model predictions in both, direction of *V*_mem_ change, and extent of *V*_mem_ change (Fig. 3 and 4). As predicted, nicotine exposure suppresses the neural brain-specific *V*_mem_ pattern, while HCN2 channel expression enhances the neural *V*_mem_ pattern and restores it even in presence of the otherwise teratogenic nicotine. Having established that HCN2 can repair the bioelectric prepattern, we next assayed its effects on brain structure and function.

### HCN2 overexpression reduces background brain defects

**Fig. 5:**
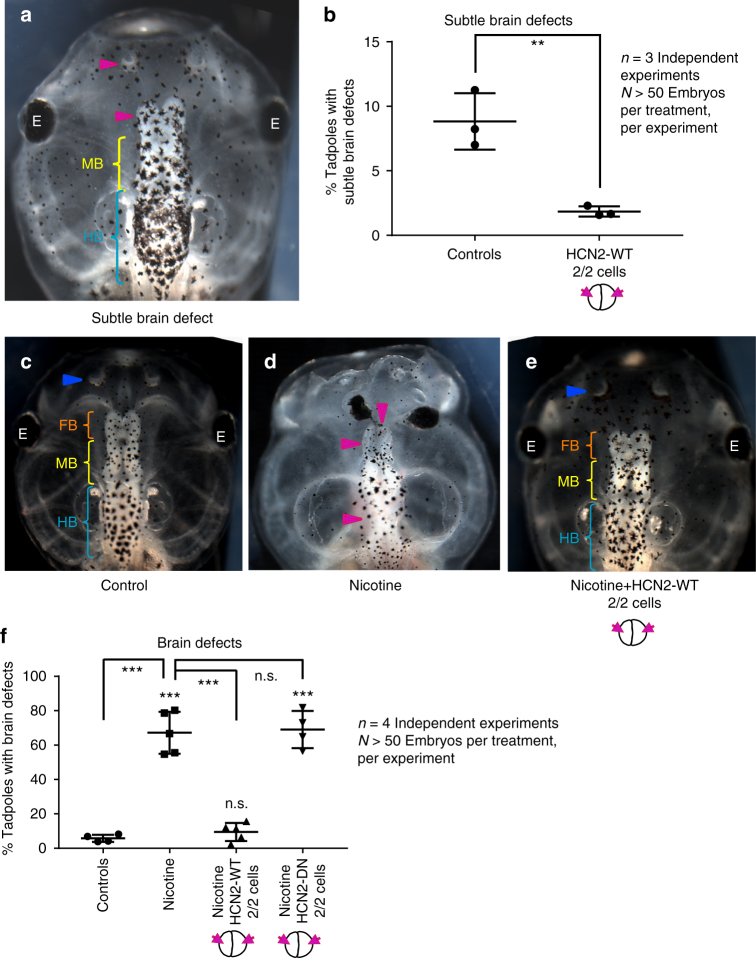
HCN2 channels rescue nicotine-induced brain morphology defects in *Xenopus* embryos. **a** Representative image of stage 45 tadpole with an example of a minor brain morphology defect showing smaller forebrain and nostril on the left (magenta arrowhead), normal midbrain (MB - yellow bracket) and normal hindbrain (HB - cyan bracket). **b** Quantification of stage 45 tadpoles for subtle changes in overall brain morphology with or without microinjecting *Hcn2-WT (wild-type)* mRNA (0.75 ng/injection) in both blastomeres at 2-cell stage as indicated in the illustrations. *Hcn2-WT* mRNA injections significantly suppress the minor brain defects seen in uninjected control embryos. Three independent experiments (*n* = 3) were conducted with *N* > 50 embryos per treatment group for each of those experiments, collected from multiple animals across independent clutches. Data were analyzed with t-test and graphed as mean ± SD, **p < 0.01. Representative images of stage 45 tadpoles: **c** control tadpole showing nostrils (blue arrowhead), forebrain (FB) indicated by the orange bracket, midbrain (MB) indicated by the yellow bracket, and hind brain (HB) indicated by the cyan bracket, **d** tadpole from embryos exposed to nicotine (0.1 mg/mL – stage 10–35) showing severe brain morphology defects as indicated by magenta arrowheads. **e** tadpole from embryos exposed to nicotine (0.1 mg/mL – stages 10–35) and microinjected with *Hcn2-WT* mRNA (0.75 ng/injection) in both blastomeres at 2-cell stage showing intact nostrils (blue arrowheads), forebrain (FB - orange brackets), midbrain (MB - yellow brackets), and hindbrain (HB - cyan brackets). **f** Quantification of stage 45 tadpoles for major brain morphology phenotypes in absence or presence of nicotine exposure (0.1 mg/mL – stage 10–35) with or without microinjection of *Hcn2-WT* or *Hcn2-DN (dominant-negative)* mRNA (0.75 ng/injection) in both blastomeres at 2-cell stage as indicated in the illustrations. A significantly high incidence of malformed brain was observed in embryos exposed to nicotine in comparison to controls. *Hcn2-WT* mRNA injection significantly reduced the incidence of malformed brain, while *Hcn2-DN* mRNA injection had no significant effect on nicotine exposure induced malformed brain. Three independent experiments (*n* = 3) were conducted with *N* > 50 embryos per treatment group for each of those experiments collected from multiple animals across independent clutches. Data were analyzed with one way ANOVA and Tukey’s post-test and graphed as mean ± SD,***p < 0.001, n.s. non-significant

As a prelude to testing its repair function, we examined the effects of HCN2 channel expression on brain morphology and patterning in normal embryos. We injected *Hcn2-WT* mRNA into both blastomeres of wild-type embryos at the two-cell stage and assayed brain morphology at stage 45 (swimming tadpoles). *Hcn2-WT* mRNA injection increased the HCN2 channel levels by ~3.1 times (310%) in the injected embryos in comparison to controls as observed by immunostaining followed by quantitative analysis of control and injected embryos (Supplementary Figure 3). Even very small instances of morphological deviation from perfectly normal brain morphology were documented, including slightly shortened nostril distances, and unequal forebrain/olfactory bulbs. In untreated control embryos, a normal basal level of minor incidences of deviation from ideal brain morphology was observed in this very strict assay (~9%). Remarkably, we noticed that *Hcn2-WT* channel-injected embryos showed a significant reduction of these minor brain morphology phenotypes (~1.5%, **p < 0.01, *t*-test *n* = 3 with *N* > 50 embryos for each group for each of the experiments collected from multiple animals across independent clutches) (Fig. 5a, b): the channel mRNA-injected embryos were better than the controls. Thus, misexpression of HCN2 channels resulted in an improvement of developmental patterning even beyond that observed in control populations of unperturbed embryos raised under optimal conditions. This result is consistent with the use of our BETSE model to identify *V*_mem_-modifying interventions that reinforce endogenous patterns and might be useful candidates for improving anatomical structure (Fig. 3c, e).

### HCN2 rescues nicotine exposure-induced brain defects

To test the predicted effect of HCN2 on nicotine’s teratogenic influences on embryonic brain patterning, we exposed *Xenopus* embryos to nicotine (0.1 mg/mL – stage 10–35). We tested the effect of both *Hcn2-WT* and *Hcn2-DN* (*dominant-negative*) construct. *Hcn2-DN* was generated by mutating the highly conserved cation selective pore-domain GYG (amino acid 402–404) to AAA, a strategy known to drastically reduce conductance and generate dominant-negative mutants of HCN4 channels^24,51^ (Supplementary Figure 2). *Hcn2-WT* or *Hcn2-DN* mRNA was microinjected into both blastomeres at two-cell stage along with nicotine exposure. Untreated (uninjected) embryos served as controls. After the embryos were developed to stage 45, we quantified brain morphology (Fig. 5c–e). Control tadpoles had correctly patterned brain tissue^5^, including normally developed nostrils, olfactory bulbs, forebrain, midbrain, and hindbrain (Fig. 5c, f). As before (Fig. 1), nicotine exposure caused a significantly high incidence of major brain morphology defects (~60%) in comparison to controls (~7%) (Fig. 5c–f). Remarkably, *Hcn2-WT* mRNA-injected embryos showed a complete rescue of brain morphology defects from nicotine exposure, to levels (~6%) indistinguishable from that of untreated control embryos (~7%) (Fig. 5f). The nicotine-exposed, *Hcn2-WT* mRNA injected tadpoles showed nostrils, olfactory bulbs and forebrain, midbrain, and hindbrain that had formed similarly to controls (Fig. 5e). In contrast, nicotine-exposed embryos injected with a mutant, dominant-negative form of HCN2 failed to show rescue of brain morphology defects (~70%) (Fig. 5f).

**Fig. 6:**
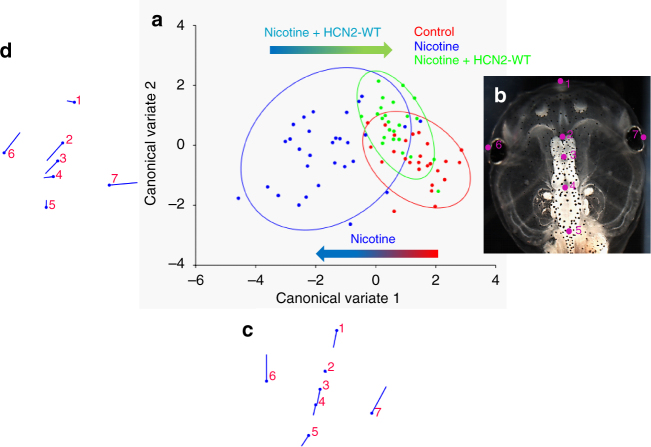
HCN2 channels restore the brain size relation to anterior head shape in nicotine-exposed embryos. Morphometrics canonical variate analysis of brain size relation to anterior head shape of stage 45 tadpoles. **a** Graphical output, showing confidence ellipses for means, at a 0.95 probability, of shape data from controls and nicotine-exposed (0.1 mg/mL – stage 10–35) tadpoles with or without *Hcn2-WT* mRNA (0.75 ng/injection) microinjected in both blastomeres at 2-cell stage. Ellipses are colored to correspond with treatment as indicated. *N* > 26 for each group. Procrustes distances show objective alteration of brain size in relation to head morphology by nicotine exposure in relation to controls as indicated by the red-blue arrow. *Hcn2-WT* mRNA microinjection along with nicotine exposure moves the brain size—head shape relation closer to controls as indicated by the blue-green arrow. **b** Stage 45 control tadpole image illustrating the 7 landmarks considered for this analysis comparing brain size and head shape. Landmarks 2, 3, 4, and 5 show the start of forebrain, transition to midbrain, transition to hindbrain, and end of hindbrain, respectively. Landmarks 1, 6, and 7 indicate the anterior most, and lateral most points of the head shape. **c** Canonical variate 1 axis legends (ball and stick diagrams) showing movement of each of the 7 landmarks in mainly anterior-posterior direction. Each ball represents the landmark as indicated by the number and the accompanying stick represents the direction and extent of movement of that particular landmark. **d** Canonical variate 2 axis legends (ball and stick diagrams) showing movement of each of the 7 landmarks in mainly lateral direction. Each ball represents the landmark as indicated by the number and the accompanying stick represents the direction and extent of movement of that particular landmark

To quantify brain shape, we used geometric morphometrics on stage 45 tadpoles^52,53^ (Fig. 6). Landmarks were chosen based on transition points between different brain regions, and lateral and anterior outermost points of the head (Fig. 6b) and recorded for *n* > 26 tadpoles for controls, nicotine, and nicotine + *Hcn2-WT* mRNA. Canonical variate analyses (Fig. 6a) were run on the data set, with Procrustes distances between each of the groups to characterize shape changes in each condition.

The quantification revealed whether the length of the brain was changed in relation to the head shape in nicotine-exposed embryos and whether *Hcn2-WT* mRNA injections restored wild-type proportions. Canonical variate analysis and Procrustes distances confirmed that control and nicotine + HCN2-WT group were not different in shape (Fig. 6a, green and red ellipses), but significantly different from nicotine-exposed embryos (blue ellipse) (Procrustes distances: Control vs. nicotine + HCN2-WT = 0.0446, Control vs. nicotine = 0.1222, and nicotine vs. nicotine + HCN2-WT = 0.1200). ANOVA of centroid shape between the controls and nicotine + HCN2-WT in relation to nicotine treatment confirmed significant differences between the groupings (*F = *12.45*, p < *0.0001). Thus, nicotine significantly changes brain shape, but *Hcn2-WT* mRNA microinjections restore brain morphology to the wild-type state.

### HCN2 restores learning in nicotine-exposed larvae

**Fig. 7:**
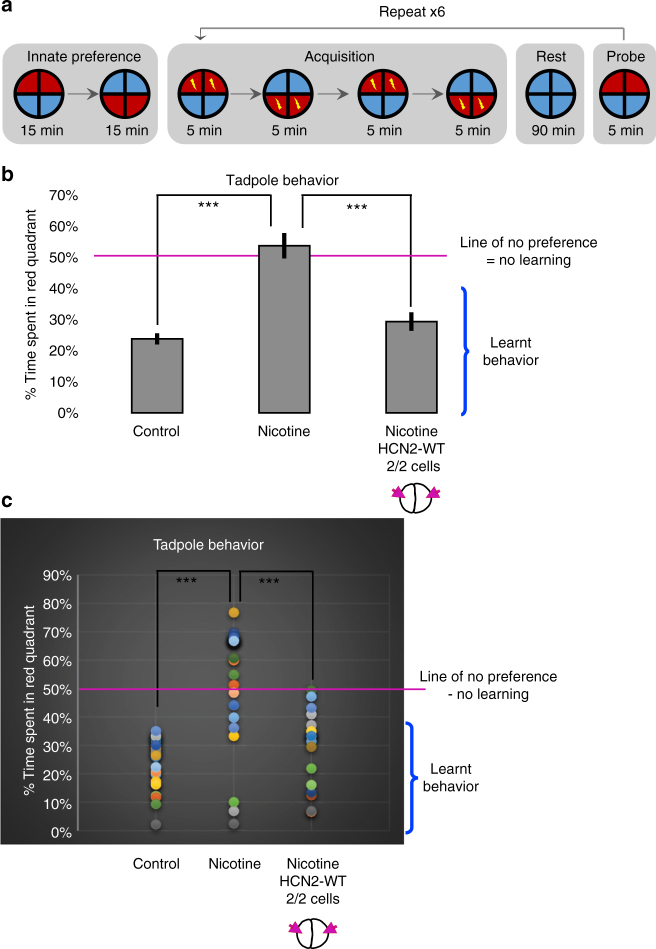
HCN2 channels restore associative learning capacity in nicotine-exposed embryos. Associative learning capacity analysis for stage 45–50 tadpoles either left untreated (controls) or exposed to nicotine (0.1 mg/mL – stage 10–35) with or without microinjections of *Hcn2-WT* mRNA (0.75 ng/injection) in both blastomeres at 2-cell stage as indicated in the illustrations. **a** The training regime used consisted of an innate preference test, a training phase, a rest period, and a learning probe. Tadpoles were placed individually in the behavior analysis robot. Motion tracking cameras under each tadpole recorded its position/behavior, and automated software executed a training cycle where animals received a shock when occupying the red half of the arena. Training, rest and testing sessions were repeated a total of six times across the trial. **b** Quantification of time spent in the red color during the final testing probe for tadpoles in each treatment group. *N* > 20 for each experimental group. Error bars indicate ± S.E.M. Data were analyzed using one way ANOVA ***p < 0.001. **c** Representation of individual tadpole’s associative learning test. *N* > 20 for each experimental group. Following the training sessions a significant red light aversion was generated in control tadpoles. Majority of nicotine-exposed tadpoles failed to demonstrate red light aversion learning during testing. Nicotine-exposed tadpoles microinjected with *Hcn2-WT* mRNA showed a restored ability to learn associative red light aversion

Using an automated behavior analysis platform^54,55^ (Fig. 7a), a robust red-avoidance behavior can be produced and quantified in normal tadpoles (Fig. 7b, c). A tadpole was classified as ‘having learned’ if their preference for red light drops below 40% of time spent in red light, averaged across the final three probe sessions of the experiment. This approach has been successfully used to quantitatively evaluate brain function and learning performance under surgical, pharmacological, and genetic manipulations^56–58^. We tested nicotine-exposed tadpoles (0.1 mg/mL – stage 10–35) with or without the *Hcn2-WT* mRNA microinjection (0.75 ng/injection) in both blastomeres at two-cell stage.

Only ~12% (*n* = 26) of the nicotine-exposed tadpoles showed learning ability; the majority failed to learn in this automated behavioral test, in comparison to control tadpoles, which showed good learning abilities (100%, *n* = 25) (Fig. 7b, c, ANOVA *p* < 0.001), even though only the able-bodied (healthy, morphologically normal) nicotine-exposed animals were tested in this assay. As expected, the brain dysmorphias caused by nicotine were accompanied by severe performance defects. However, nicotine-exposed embryos receiving *Hcn2-WT* mRNA showed significant improvement in learning ability (85%, *n* = 20) in comparison to nicotine-exposed tadpoles (Fig. 7b, c, ANOVA, *p* < 0.001). Thus, HCN2 channels not only repair morphology, but also restore cognitive learning abilities in nicotine-exposed tadpoles.

### HCN2 restores marker expression in nicotine-treated embryos

To determine the effects of HCN2 channel intervention in the hierarchy of known transcriptional regulators of brain patterning, we analyzed the expression of the key transcription factors *otx2* (forebrain and midbrain), *emx* (telencephalon and forebrain), *xbf1* (forebrain), and *Pax6* (forebrain and eye)^59–62^.

**Fig. 8:**
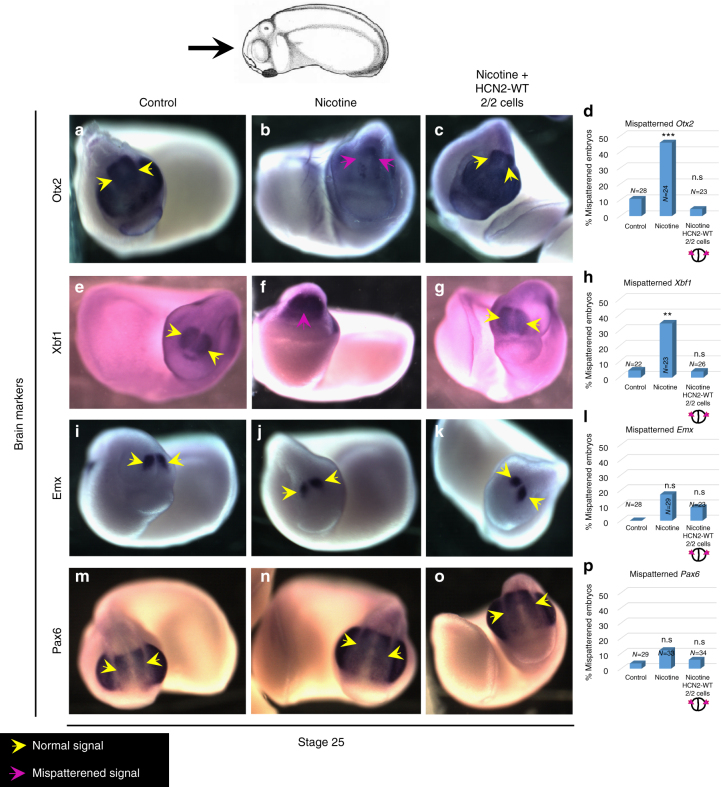
HCN2 channels restore nicotine exposure induced mispatterning of brain markers during neural development. Stage 25 embryos as illustrated with the angle of view marked by the black arrow. Control (untreated/uninjected) embryos (**a**,** e**, **i**,** l**), embryos exposed to nicotine (0.1 mg/mL – stage 10 onwards) **(b**,** f**,** j**, **n)**, and nicotine-exposed embryos microinjected with *Hcn2-WT* mRNA (0.75 ng/injection) in both blastomeres at 2-cell stage (**c**, **g**, **k**, **o**). In situ hybridization for *Otx2* (**a**–**d**), *Xbf1* (**e**–**h**), *Emx* (**i**–**l**), and *Pax6* (**m**–**p**) show that nicotine exposure leads to significantly mispatterned expression (magenta arrows) of *Otx2* [46% *n* = 24] and *Xbf1* [35% *n* = 23], but has little effect on *Emx* [17%, *n* = 29] and *Pax6* [12%, *n* = 33] in comparison to controls [10%, *n* = 28, 4.5%, *n* = 22, 0%, *n* = 28, and 3%, *n* = 29, respectively]. Nicotine-exposed embryos that were also microinjected with *Hcn2-WT* mRNA showed largely normal expression of *Otx2* [4% *n* = 23], *Xbf1* [4% *n* = 26], *Emx* [9% *n* = 23], and *Pax6* [6% *n* = 34] in comparison to controls

Embryos with or without *Hcn2-WT* mRNA microinjection (both blastomeres at 2-cell stage) were exposed to nicotine from stage 10. Uninjected and untreated embryos were used as controls. Embryos were analyzed at stage 25 for expression of *otx2, xbf1, emx*, and *pax6* by in situ hybridization (Fig. 8). The nicotine-exposed embryos showed significantly reduced expression of *otx2* (in both area and intensity) and reduced or mispatterned expression of *xbf1* in significant number of embryos [46% (χ^2^, *p* < 0.001) and 35% (χ^2^, *p* < 0.01), respectively] in comparison to control embryos (10% and 4.5%, respectively). In marked contrast, *Hcn2-WT* mRNA-injected embryos showed normal pattern of *otx2* and *xbf1* expression at similar levels to that of controls (4 and 4%, respectively). Nicotine exposure had little effect on *emx* and *pax6* expression pattern, with minor reductions in a few embryos (17 and 12%, respectively) in comparison to controls (0 and 3%, respectively). *Hcn2-WT*-injected embryos exhibited normal *emx* and *pax6* expression pattern at similar levels to controls (9 and 6%, respectively). Thus, HCN2 channel rescue of nicotine-induced defects includes correction of *otx2* and *xbf1*, connecting HCN2-mediated repair of brain to the known molecular-genetic cascade of brain patterning factors.

## Discussion

Nicotine is a well−studied developmental neuroteratogen in humans^29^, non-human primates^31^, and invertebrates^32^. A fundamental gap in this area is the lack of mechanistic knowledge of how the effects on individual cell physiology result in impaired cognitive function. We addressed the developmental events that underlie the middle layer between cell behavior and organismal behavior—teratogenic effects of nicotine exposure on morphogenesis of the developing brain.

Bioelectric circuit^9–11,63^ dynamics are distinct from those of chemical gradients^64–66^ and are often complex. To bridge the gap between cell-level molecular electrophysiology and organism-level patterning and behavior, we used BETSE,^41^ a powerful and flexible biorealistic platform that enables predictions of bioelectrical patterns from channel expression and function data. We built a model of bioelectric patterns of neural stage *Xenopus* embryo, taking into account spatiotemporal dynamics of all different parameters including gap junctions, tight junctions, and relevant biochemical signals.

The endogenous early embryonic *V*_mem_ patterns across the neural tube are required for normal brain development^28^. Nicotine, an ion channel ligand, disrupts brain patterning (Fig. 1). We analyzed the BETSE model to understand how nicotine and IT actions would integrate at the level of embryonic *V*_mem_ patterning, and generate predictions and testable hypotheses.

Nicotine exposure diminished the endogenous neural tube hyperpolarization in the model (Fig. 3). Such disruptions in the brain specific *V*_mem_ pattern have already been shown to cause serious brain morphology defects^28^. In the model, HCN2 channels were seen to behave in a unique context-specific manner (Fig. 3f) that specifically amplifies the differential neural tube *V*_mem_ pattern across the neural plate despite the presence of nicotine (fitting the functional criteria of the model-derived IT). This predicts that context-specific action of HCN2 would strengthen the *V*_mem_ pattern otherwise diminished by nicotine and thus restores downstream transcription factors and brain patterning. Precisely, this was observed in our physiological profiling experiments (Fig. 4). It is also important to note that the model predicts that due to its context specific action, HCN2 channels also enhance the brain specific *V*_mem_ pattern of control (non-nicotine exposed) embryos (Fig. 3), consistent with our observation of patterning improvement of even the low incidence of brain defects in control batches of embryos (Fig. 5b). The BETSE model proved essential to quantitatively test this hypothesis and evaluate the properties of HCN2-containing circuits to reveal that they can indeed overcome the effects of nicotine on *V*_mem_ and do so appropriately depending on location in the neural tube center and its lateral areas. The balance of *V*_mem_ contrast between the neural tube and surrounding tissue has been shown to control the balance of proliferation and apoptosis in neural tube development^48^. This kind of sharpening of boundaries between distinct bioelectric compartments explains why HCN2 channels are able to correct even background brain defects (Figs. 5a, b).

An alternative case is where nicotine may directly affect endogenous HCN2 channels^46^, resulting in brain patterning defects. This suggests that normal endogenous HCN2 activity is required for proper brain patterning. However, our experiments with dominant-negative HCN2 channel shows that native HCN2 channel activity is not required for proper brain patterning (Supplementary Figure 4), thus negating this possibility. The effect of nicotine on the embryonic *V*_mem_ pre-pattern through nAChR (Figs. 2 and 3) is sufficient to explain the observed change in bioelectric state and the subsequent phenotypes, but our data do not rule out additional endogenous targets of nicotine. This is consistent with bioelectric mechanisms of action suggested for a number of other teratogenic chemicals^67^ and genetic syndromes^68–70^.

A critical next step for developmental physiology is to extend the understanding of single-cell electrophysiological properties (and ion channel expression) to spatialized dynamics that can explain the maintenance or disruption of large-scale patterns with relevance to developmental or regenerative patterning. To test predictions of the BETSE model, we generated *V*_mem_ maps of neurula stage *Xenopus* embryos under the different treatment conditions (Fig. 4). As predicted by the BETSE model, nicotine treatment indeed diminished the *V*_mem_ pattern (depolarized) by ~15 mV (peak *V*_mem_ in the neural tube) and HCN2 channel even in presence of nicotine increased the *V*_mem_ pattern (restored hyperpolarization) by ~35 mV (peak *V*_mem_ in the neural tube) (Figs. 3 and 4). In the case of HCN2 channel alone, the observed enhancement of *V*_mem_ pattern in relation to controls (~15 mV) was much less than the predicted value of increase by ~30 mV (Figs. 3 and 4). Since HCN2 channels are also regulated by cAMP and the context of cell metabolism, this difference may be the result of as yet unknown aspects of cell metabolism. It is also possible that there are other ion channels and pumps at play within the cells, affecting the action of HCN2 or directly influencing *V*_mem_. We found an excellent quantitative match between the BETSE model’s predictions and the observed physiological and functional outcomes. To our knowledge, this is the first use of computational modeling to reveal spatialized bioelectric signaling dynamics that not only correctly predict the effects of a teratogen, but also show how to implement a rescue strategy.

Bioelectric interventions targeting ion flux have been used to induce regenerative response^16,17^. Here, we established a proof-of-principle extending this strategy to the use of ion channels as a reparative intervention for birth defects. Remarkably, HCN2 overexpression in embryos exposed to nicotine led to a complete rescue of nicotine-induced brain morphology defects (Fig. 5c–f). HCN2 channel overexpression restored not only gross brain morphology, but also the normal relation between brain length and the shape and size of the head (Fig. 6). This suggests that HCN2-mediated rescue involves sensing information from the surrounding regions to develop a brain with the correct size in relation to surrounding tissues and overall embryo proportions. Our prior work had pointed to the existence of a mechanism that coordinates brain sizing with remote tissue regions^48^. These consequences of HCN2 function are most likely due to its ability to act in a context-specific manner (Figs. 3 and 4)—it does not merely bring every cell to the same *V*_mem_, but effects specific changes of resting potential that are different in distinct regions based on the local pre-existing *V*_mem_. This ability to reinforce spatial differences enables embryos to establish the crucial embryonic brain-specific *V*_mem_ pre-pattern^28,48^ that is otherwise erased by nicotine exposure (Fig. 4).

A notable finding was the ability of HCN2 channels to improve the brain morphology of control embryos (Fig. 5a, b). This effect is likely also attributable to the context-specific actions of HCN2, which significantly enhance the crucial embryonic brain’s *V*_mem_ pre-pattern of these embryos (Figs. 3 and 4). These observations suggest that the restorative action of HCN2 is not limited to nicotine-induced brain morphology defects, but could reveal a more general mechanism of action, reinforcing the endogenous bioelectrical signals required for correct developmental brain morphology that might be partially destabilized by a wide range of exogenous teratogens or endogenous physiological/genetic lesions. The full scope of the kinds of defects that can be rescued by HCN2 remains to be delineated and is an active area of investigation in our laboratory.

We tested the effects of HCN2 on learning behavior in nicotine-exposed *Xenopus* embryos using an automated training apparatus^54–56^. Our data showed that HCN2 channels largely restore learning abilities in nicotine-exposed tadpoles (Fig. 7). This suggests that HCN2 along with restoring the physiological states necessary for normal brain morphology (Figs. 5 and 6) restores the internal wiring and networks necessary for normal learning abilities (Fig. 7). Given that HCN2 is able to fix learning abilities in addition to gross brain morphology, we suggest that HCN2 or other *V*_mem_-based therapies could represent an important new direction for addressing learning and memory disorders that are related to such defects.

Endogenous *V*_mem_ patterns in neurula stage *Xenopus* embryos are critical for proper patterning and sculpting of brain tissue. Here we established an amphibian model for understanding nicotine neuroteratogenesis at the level of gene expression, electrophysiology, morphology, and behavior. Because of the central role of bioelectric circuits in normal brain development and teratogenesis, we formulated a predictive, biorealistic model of developmental physiology that explained the patterning of resting potentials across the neural tube under wild-type and disease conditions. Our model is the first quantitative explanation of teratogenesis targeting bioelectric controls of growth and form. Most importantly, it predicts that reagents that sharpen borders between bioelectric compartments will reinforce normal patterning, thus identifying a context-sensitive HCN2 ion channel as an intervention tool. We used HCN2 to confirm this prediction in vivo. Forced expression of HCN2 mRNA rescued nicotine-induced malformations, restoring not only the anatomy, but the gene expression in the nascent brain, and ultimately improving behavioral metrics of learning and memory. These data identify a patterning function that can be implemented using HCN2 in synthetic biology or in vivo. Taken together, our data shed light on a new mechanism of brain defects and establish proof-of-principle for using developmental physiology models to understand disorders of pattern and test interventions in silico. We provide a tool for predictive bioelectric modulation and demonstrate its use as a part of a tractable strategy for manipulating neurogenesis and neural patterning. Future work targeting native channels with small molecule compounds to effect rescue without gene therapy are currently on-going in our lab. Thus, activation or expression of specific channels is a general strategy for biomedical approaches to complex organ patterning in the context of birth defects, regenerative medicine, and bioengineering.

## Methods

### Animal husbandry

*Xenopus laevis* embryos were fertilized in vitro according to standard protocols^71^: in 0.1 × Marc’s Modified Ringer’s (MMR; 10 mM Na^+^, 0.2 mM K^+^, 10.5 mM Cl^-^, 0.2 mM Ca^2+^, pH 7.8). *Xenopus* embryos were housed at 14–18 °C (14 °C overnight after injection and subsequently at 18 °C), and staged according to Nieuwkoop and Faber^72^. We saw the normal very-low background levels of abnormal morphologies (<9%), indicating the good health of our animals and good rearing conditions. For animals used in behavior trials, individuals were raised under 12 h:12 h light:dark cycle at a temperature of 16 °C, at no more than 30 individuals per 100 × 25 mm Petri dish. After stage 46, tadpoles were fed twice per day on standard sera micron powdered food until behavioral testing. All experiments were approved by the Tufts University Animal Research Committee (M2017-53) in accordance with the guide for care and use of laboratory animals.

### Microinjections

Capped synthetic mRNAs generated using mMessage mMachine kit (Ambion) were dissolved in nuclease free water and injected into embryos immersed in 3% Ficoll using standard methods^71^. Each injection delivered between 0.5–1 ng of mRNA (per blastomere) into the embryos, at the indicated stages into the middle of the cell in the animal pole. *Hcn2-WT* and *Hcn2-DN* were mammalian (mouse) (HCN) channel 2, modified as detailed in the supplemental documents.

### Drug exposure

*Xenopus* embryos were incubated in chemicals or pharmacological blockers dissolved in 0.1 × MMR during the stages of interest as indicated in respective experiments followed by several washes with 0.1 × MMR. The embryos were exposed (from stage 10 to 35 unless otherwise specified, because neural/brain tissue development takes place in this time period, allowing specific testing of effects on these processes while allowing cleavage and gastrulation to proceed normally) to the following: 0.1 mg/mL nicotine (Sigma). The dose of nicotine was titrated to a level at which no general toxicity was observed and survival rate of embryos was similar to untreated controls.

### Morphometrics

Tadpoles used for morphometric analysis were imaged with a Nikon SMZ1500 microscope with a Retiga 2000R camera and Q-capture imaging software. Landmark data were then recorded using ImageJ software^73^. Landmarks for morphometric analysis were chosen based on biological relevance and reproducibility across tadpoles with varying brain and head morphologies. Landmarks were: (1) anterior most tip of the head; (2) between the two olfactory bulbs at the beginning of the forebrain; (3) laterally center of the brain at the point of transition between forebrain and midbrain; (4) Laterally center of the brain at the point of transition between midbrain and hindbrain; (5) Transition point between hindbrain and spinal cord; and (6) and (7) Lateral outermost points of the head along the eyes. MorphoJ software^74^ was used for Canonical Variate Analysis, to quantify and graphically represent change in brain regions in relation to head shape. MorphoJ software was also used to calculate Procrustes distances and perform statistical analysis. Our analysis is conservative as nicotine-treated embryos that had major portions of their brain missing were not used since the same landmarks as controls could not be used (e.g., Fig. 1c). Only those tadpoles that had brain morphology defects, but still could be used to place the landmarks were used (e.g., Fig. 5d).

### Associative learning behavior test

All behavioral trials were performed with a custom built automated training robot^54,56^. The device consists of an array of 12 individual chambers, each capable of holding a 60 × 15 mm petri dish filled 15 mls of 0.1 × MMR. Below each dish is a machine vision camera (Insight-Micro 1400, Cognex Corporation, Natick, MA, USA), which uses a background subtraction algorithm to track any animals in the chamber in a Cartesian manner. Illumination is provided to each chamber independently from above, by a control module capable of specifying color and intensity by quadrants within each chamber. Red or blue light is delivered by light-emitting diodes (Osram Semiconductors, blue LED; 470 nm part no. LBW5SM, red LED, 635 nm part no. LRG6SP) and *Xenopus* see both given the spectral profiles of their three known cone classes^75^. Each chamber also has a set of six, equidistant, iridium oxide-coated titanium electrodes allowing the delivery of mild to strong electric shocks. All shocks delivered during wavelength-mediated training experiments were 1.2 mA AC currents, pulsed for 100 ms followed by 300 ms of no shock. This value was previously determined to be the lowest that elicits a behavioral response and no animals displayed physiological or behavioral abnormalities upon completion of testing.

Tadpole color-based associative learning assay was performed as documented before^56^ (Fig. 7a). Individual tadpoles were introduced to the chamber, which was illuminated with half red and half blue light, in absence of any punishment to probe their innate color preferences. Innate testing lasted 30 mins with colors across quadrants inverted after 15 mins to avoid considering stationary tadpole as having a 100% preference for a particular color (if the tadpole is stationary inverting the color would result in 50/50 preference). Tadpole then enters learning acquisition phase where tadpole receives a 1.2 mA shock if it occupies the red quadrant. This duration is 20 mins, with colors in the chamber being inverted every 5 mins. Then tadpoles are given a 90 min rest period where the entire chamber is illuminated with blue light and no shock punishment is delivered. Finally, the tadpole is probed for learning of light preference by giving them a choice between red and blue light for 5 mins with no punishment. The entire block of acquisition-rest-probe is repeated 6 times.

All tadpoles used in behavioral trials were stage ~48. A tadpole was determined to have learned if their preference for red light was below 40%, averaged across the final 3 probe sessions of the experiment. Note, that tadpoles were fed directly before trials and small amount of food was added to each chamber during training, as hungry tadpoles fail to learn.

### In situ hybridization

*Xenopus* embryos were collected and fixed in MEMFA (1 hour at room temperature)^71^ and in situ hybridization was performed as previously described^71^. Briefly, the embryos were washed with PBS 0.1% Tween 20 (PBST) and transferred through series of methanol washes 25–50% to 75–100%. In situ antisense probes were generated in vitro from linearized templates using a DIG-labeling mix (Roche). Chromogenic reaction times were optimized for signal-to-background ratio. Probes used were *otx2* (ref. ^76^), *xbf1*, and *emx* (refs. ^77,78^). Antisense probe for *Xenopus* HCN2 was generated from *X. laevis* hcn2.L IMAGE clone 5514485 (Dharmacon): a HindIII fragment was deleted, leaving exons 2–4 and part of exon 5 as probe^79^.

### Imaging Vmem using CC2-DMPE:DiBAC4(3)

CC2-DMPE and DiBAC_4_(3) voltage reporter dyes were obtained from Invitrogen and used as per the standard protocol, including dark-field and flat-field correction^63^. Briefly, the use of two dyes with opposite emission profiles simultaneously provides an internal control and allows ratiometric normalization. CC2-DMPE stock (5 mM) was dissolved 1:1000 in 0.1 × MMR and the embryos were incubated in the dark in this solution for at least 1 h followed by 5 washes with 0.1 × MMR. DiBAC_4_(3) stock (1.9 mM) was dissolved 1:1000 in 0.1 × MMR and the CC2-DMPE-stained embryos were then incubated in the dark in this solution for at least 30 min washed thoroughly in 0.1 × MMR, followed by visualization under the microscope. An Olympus BX-61 microscope equipped with a Hamamatsu ORCA AG CCD camera, and controlled by MetaMorph software (Molecular Devices), was used to collect signal. NIH ImageJ software was used to quantify the fluorescence intensities of the CC2-DMPE:DiBAC_4_(3) signal.

### Intracellular recordings from embryo cells

Membrane potentials were measured using an oocyte clamp OC-725C amplifier (Warner Instruments) with a single voltage electrode. Microelectrodes were made from thin-walled borosilicate glass pulled with a flaming/brown micropipette puller (p-97; Sutter Instruments) and back-filled with electrode solution (2 M potassium acetate, 10 mM KCl, and 5 mM HEPES, pH 7.5). Tip resistances were 80–100 MΩ. Electrode penetration of ectodermal cells was by visual guidance on a fixed-stage microscope (Zeiss) using a three axis micromanipulator.

### Immunofluorescence

HCN2 channel detection was performed by immunofluorescence for HCN2 channel. Briefly, embryos were fixed overnight in MEMFA at 4 °C (ref. ^71^). The embryos were permeabilized in PBS 0.1% Triton X-100; blocked with 10% goat serum in PBST for 1 hour at room temperature; and incubated at 4 °C overnight with primary antibody (Anti-HCN2: ThermoFisher Scientific PA1-918) at 1:500 dilution in PBST + 10% goat serum (blocking buffer). Embryos were washed six times in PBST and incubated with Alexa Fluor-conjugated fluorescent secondary antibody (Invitrogen) at 1:500 dilution in PBST + 10% goat serum overnight at 4 °C. Sections were washed six times and photographed using an Olympus BX-61 microscope equipped with a Hamamatse ORCA AG CCD camera, and controlled by MetaMorph software. NIH ImageJ software was used to quantify the fluorescence intensities of the immunostained embryos.

### Molecular biology

A mouse HCN2 cDNA clone was kindly provided by Dr. Jeanne Nerbonne (Washington University). The HCN2 cDNA was cloned into the eukaryotic expression vector pcDNA3 (Thermo Fisher Scientific). To create the dominant-negative (DN) HCN2 mutation, the entire GYG signature motif in the channel pore was mutated to alanine-alanine-alanine (AAA) by site-directed mutagenesis. The forward primer was: 5’-ATGAGCCACATGC TGTGTATCGCCGCGGCACGACAAGCACCCGAGAGCATGACA-3’ and the reverse primer was: 5’-TGTCATGCTCTCGGGTGCTTGTCGTGCCGCGGCGATACA CAGCATGTGGCTCAT-3’. The WT or DN mutant of HCN2 was then sub-cloned into a bi-cistronic mammalian expression vector, pIRESGFP1 (kindly provided by Dr. David Johns, Johns Hopkins University), to create a CMVp-HCN2-IRES-eGFP fusion for subsequent studies. The HCN2 channel construct function was tested using voltage clamping in HEK293 cells (which do not have endogenous HCN channels) (Supplementary Fig. 2A).

### Cell culture and transfection

HEK293 cells were cultured in Dulbecco’s Modified Eagle’s Medium supplemented with 10% fetal bovine serum, 100 μg/ml penicillin, and 100 μg/ml streptomycin in a 37 °C, 5% CO2, 95% air incubator as previously described (Makielski et al. 2003). Approximately 1 × 105 cells were seeded in a 35-mm-diameter culture dish containing 1.5-ml culture media. A volume of 0.7 µg DNA of HCN2-WT or HCN2-AAA or the empty vector control was transiently transfected into HEK293 cells at 80% confluency using a Fugene6 transfection kit (Roche Applied Science, Indianapolis, IN) following manufacturer’s instructions. The transfected cells were incubated at 37 °C for 24 h to allow protein expression.

### Voltage clamp recordings

Whole-cell HCN2-encoded inward currents (*I*_HCN_) were recorded at room temperature from transfected HEK293 cells using voltage clamping as previously described (Ye et al. 2008; Ye and Nerbonne 2009). The extracellular solution contained in (mM): NaCl 110, MgCl_2_ 0.5, KCl 30, CaCl_2_ 1.8, and HEPES 5 while the pipette solution contained (in mM): NaCl 10, MgCl_2_ 0.5, KCl 130, HEPES 5, and EGTA. The cells showing green fluorescence were identified under epifluorescence and selected for *I*_HCN_ recordings. Recording electrodes were fabricated from borosilicate glass (WP Instruments) on a two stage (P-87; Sutter Instrument) vertical puller. *I*_HCN_ was evoked in response to hyperpolarizing voltage steps to potentials between −60 and −130 mV from a holding potential of −40 mV. Data were collected using an Axopatch 200B amplifier interfaced to a Digidata1322A acquisition system (Molecular Devices, Sunnyvale, CA). Data were subsequently processed and analyzed using Axon pCLAMP10 Software Suite (Molecular Devices, Sunnyvale, CA). A one-way ANOVA with Bonferroni correction was carried out to determine the statistical significance between experimental groups. *P* < 0.05 was considered a significant difference between two groups.

### Statistics

All statistical analyses were performed using GraphPad Prism7. At least three independent experiments (*n* > 3) were conducted with *N* > 50 embryos for each treatment groups for each of those experiments collected from multiple animals across independent clutches. Data were analyzed by *t-*test (for two groups) or ANOVA (for more than two groups), as indicated with each experiment.

### Data Availability

All data generated or analyzed during this study are included in this published article and its supplementary information files, or from the corresponding author upon request.

## Electronic supplementary material

Supplementary Information
