# Supplementary material for: HCN2 Rescues brain defects by enforcing endogenous voltage pre-patterns
Source: Nat Commun. 2018 Mar 8;9:998. doi: 10.1038/s41467-018-03334-5 (PMC5843655; doi:10.1038/s41467-018-03334-5)
Supplement: Supplementary file 1 — Supplementary Information [file 41467_2018_3334_MOESM1_ESM.pdf]

HCN2 Rescues Brain Defects by Enforcing Endogenous Voltage Pre-patterns

Pai *et al.*

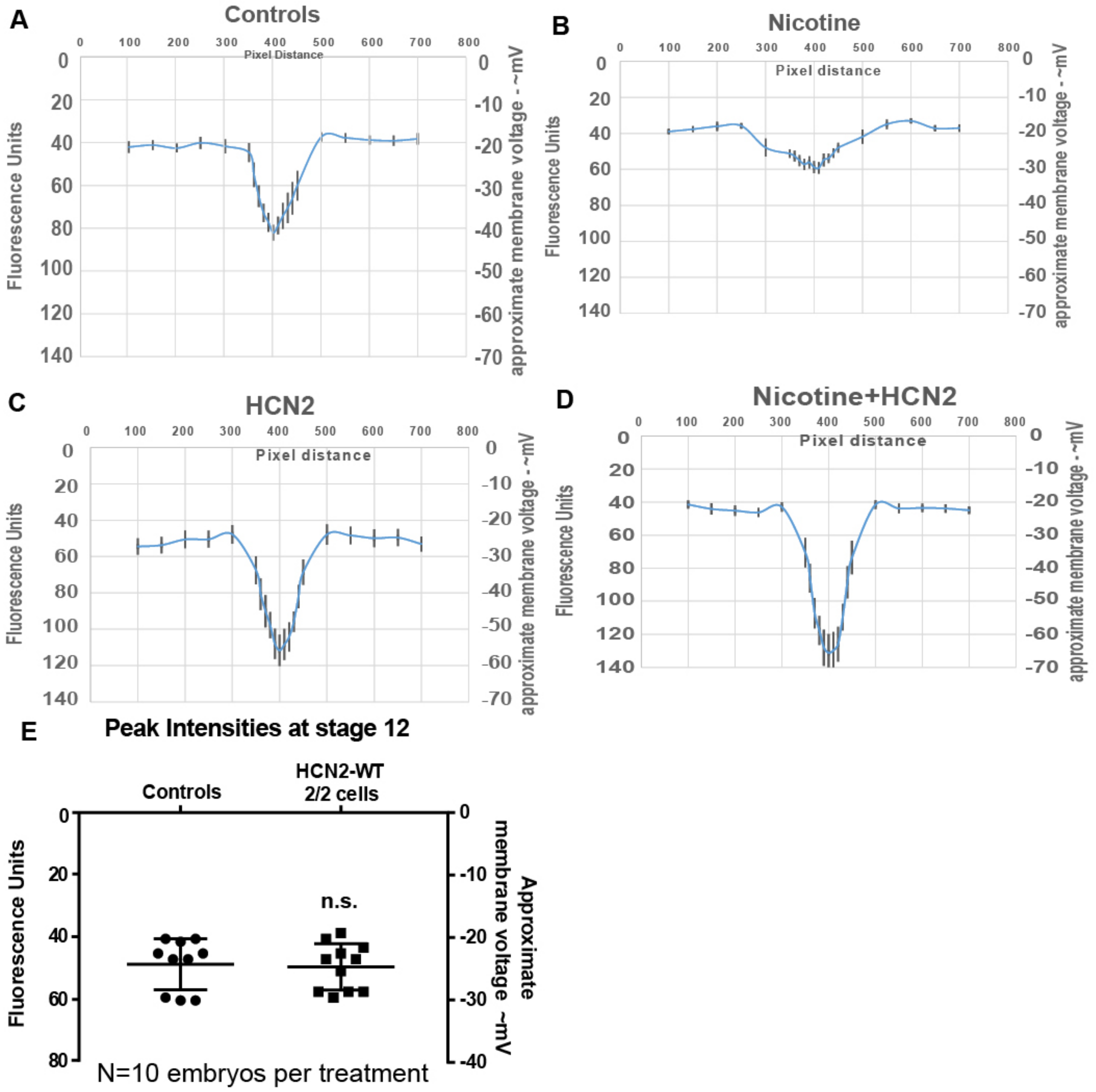

Supplementary Figure 1: Voltage reporter dye imaging and electrophysiology mediated voltage estimations of *Xenopus* embryos. **(A)** Quantification of voltage reporter dye images (CC2-DMPE:DiBAC$_4$(3)) of stage 15–17 *Xenopus* embryos along the red dotted line as indicated in Figure 4E, along with electrophysiology based membrane voltage approximations (as previously reported in Refs. 27 & 28. Embryos were either, untreated (controls) **(A)**, exposed to nicotine (0.1mg/mL – stage 10-35) **(B)**, microinjected with *Hcn2-WT* mRNA (0.75ng/injection in both blastomeres at 2-cell stage) **(C)**, or exposed to nicotine as well as microinjected with *Hcn2-*

WT mRNA **(D)**. N=10 for each treatment group at each of the indicated spatial distance in pixels. Data were analyzed using One Way ANOVA. **(E)** Quantification of peak fluorescence intensity and electrophysiology based membrane voltage approximations (as previously reported in Refs. 27 & 28) from voltage reporter dye images (CC2-DMPE:$DiBAC_4(3)$) of stage 12 *Xenopus* embryos within the neural tube at the intersection of the red and black dotted lines in the inset illustration in Figure 4E. At stage 12, there is no difference in the neural tube peak intensities between the controls and *Hcn2-WT* mRNA (0.75ng/injection in both blastomeres at 2-cell stage) injected embryos. N=10 for each group was collected multiple animals and independent clutches.

Data were analyzed using t-test and graphed as mean ± SD; n.s. = not significant.

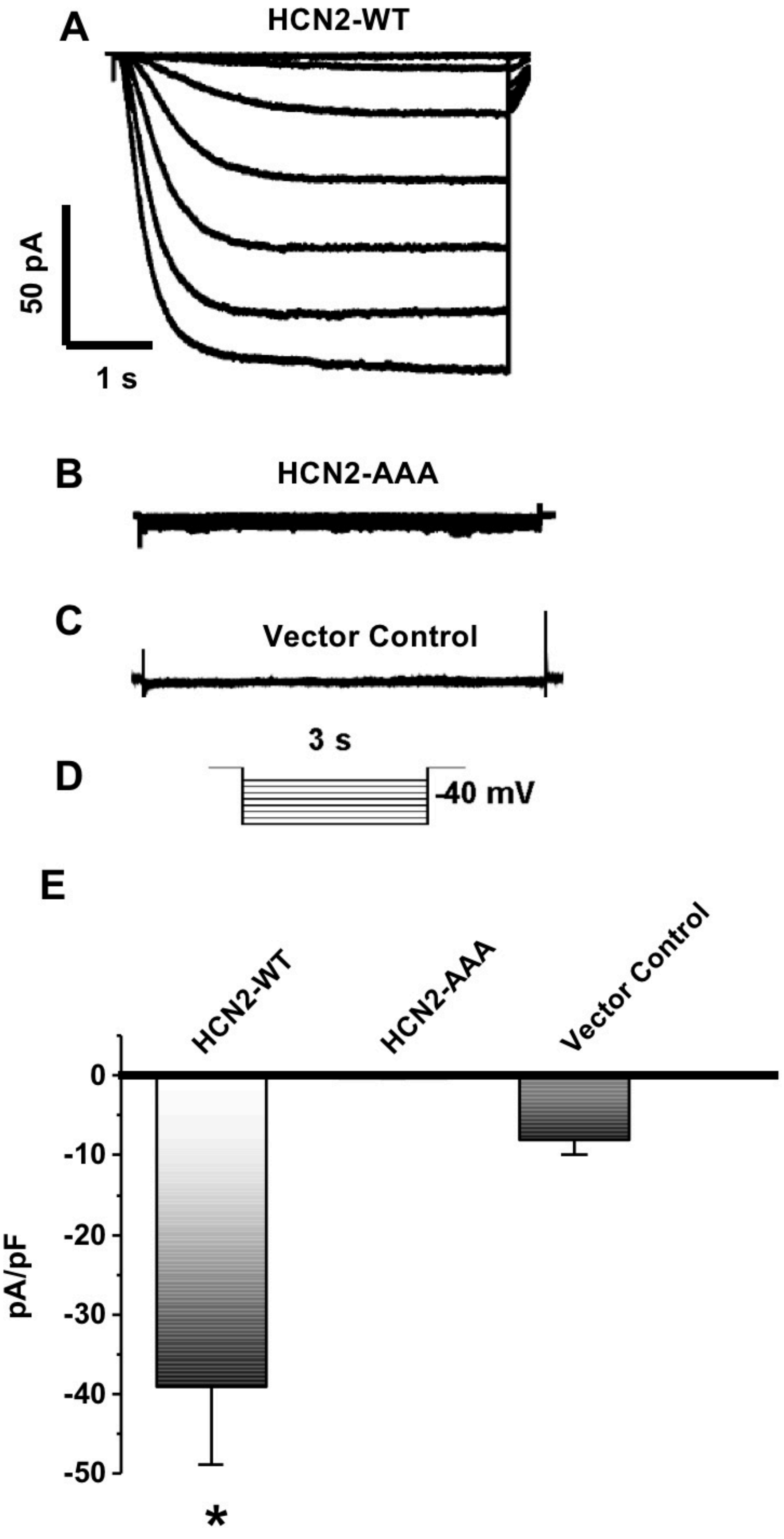

A
HCN2-WT
50 pA
1 s
B
HCN2-AAA
C
Vector Control
3 s
D
-40 mV
E
HCN2-WT
HCN2-AAA
Vector Control
0
-10
-20
-30
-40
-50
pA/pF
*

Supplementary Figure 2: Dominant-negative effects of HCN2-AAA in HEK293 cells.
Representative HCN current traces recorded from HEK293 cells expressing *HCN2-WT* **(A)**, *HCN2-AAA* **(B)**, and empty vector control **(C)**. I_{HCN} (HCN current) was elicited by recording protocol **(D)** containing stepwise hyperpolarization to -120 mV from holding potential of -40 mV and normalized to membrane capacitance. Summary data for peak I_{HCN} from HEK293 cells expressing *HCN2-WT*, *HCN2-AAA*, and vector control is shown in **(E)**.
One way ANOVA, *$p<0.05$ (n=4).

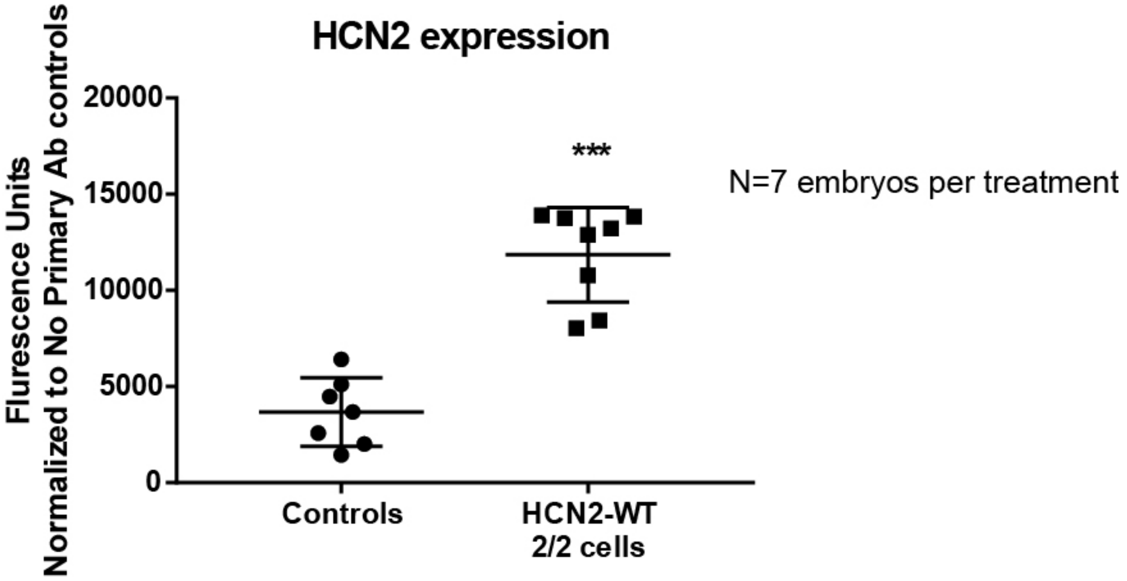

Supplementary Fig. 3: HCN2 expression levels in Hcn2-WT injected embryos. Quantification of HCN2 immunostained stage ~12 *Xenopus* embryos which were either left uninjected (controls) or injected with *Hcn2-WT* mRNA in both blastomeres at two-cell stage. *Hcn2-WT* injected embryos show a significantly high level of HCN2 channel expression (~3.1 times/310%). Embryos were obtained from multiple animals across independent clutches. Immunostaining was quantified using ImageJ software.
Data were analyzed by t-test and plotted as mean ± SD; ***-p<0.001.

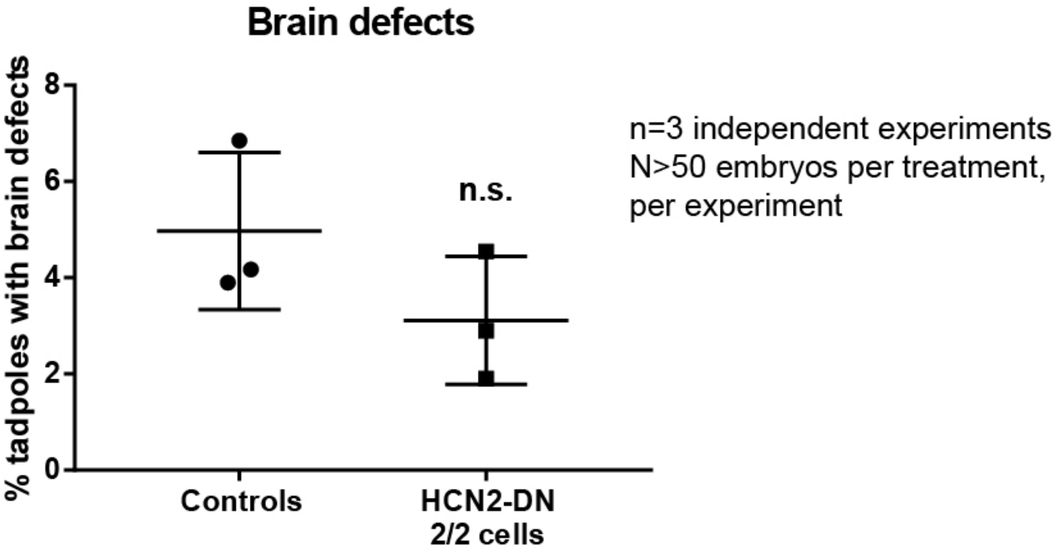

Supplementary Fig. 4: Hcn2 Dominant-negative mRNA has no effect on brain patterning. Quantification of stage 45 tadpoles for major brain morphology phenotypes in uninjected (controls) and *Hcn2-Dominant-negative* (*Hcn2-DN*) injected (in both blastomeres at two-cell stage) embryos. No significant increase in incidence of malformed brain was observed in embryos injected with *Hcn2-DN*. Three independent experiments (n=3) were conducted with N>50 embryos per treatment group for each of those experiments collected from multiple animals across independent clutches.

Data were analyzed with t-test and graphed as mean ± SD; n.s. = not significant.

Supplementary Note 1: Description of Computational Modeling

Modeling Fundamental Bioelectricity

Bioelectrical modeling utilized the **B**io**E**lectric **T**issue **S**imulation **E**ngine (BETSE), a finite volume based simulator specially designed to study bioelectrical dynamics [1]. BETSE software and associated documentation is available from:

https://gitlab.com/betse/betse

The core BETSE algorithm handles bioelectric signaling from a molecular perspective, which involves calculating all components of ion flux across membrane segments of a cell, calculating divergence of net flux across membranes to update concentrations in cells, calculating ion currents and charge in terms of net flux and concentration of ions in cells, and determining V_{mem} in terms of net charge density at the membrane. All simulations included the ions: Na^{+}, K^{+}, Ca^{2+}, Cl^{-}, HCO_3^{-}, H^{+}, and non-membrane permeable anionic proteins modeled as P^{-}. Concentrations of ions (and all BIGR network substances) were defined in both intracellular and extracellular regions, with initial concentrations as summarized in supplementary Table 1.

The fundamental elements and aspects of bioelectric phenomena are reviewed in [1–3]. ATP-powered ion pumps such as the ubiquitous sodium-potassium adenosine triphosphatase ion pump (Na^{+}/K^{+}-ATPase), which moves 3 Na^{+} from cell to extracellular space and 2 K^{+} from extracellular space to the cytosol for each ATP molecule that is hydrolysed, are instrumental in creating electrochemical gradients of Na^{+} and K^{+} across the membrane. In most metazoan cell types, the Na^{+} electrochemical gradient created by Na^{+}/K^{+}-ATPase activity favors movement of Na^{+} into cells, and therefore depolarization of V_{mem} with increased membrane permeability to Na^{+} ions ($P_{mem}\,Na$), whereas the K^{+} electrochemical gradient favors movement of K^{+} out of cells, and therefore hyperpolarization of V_{mem}, with increased membrane permeability to K^{+} ions ($P_{mem}\,K$). Transmembrane voltage (V_{mem}) ultimately arises from net ion charge density across the membrane, where the membrane acts as a capacitor. Therefore, in simulations, V_{mem} was calculated from an initial state of 0.0 and zero net charge in cells using the net ion current density across the membrane (J_{mem}) in proportion to the the patch capacitance of the membrane ($C_{mem} = 0.05\,F/m^2$), via the simple expression relating the change in voltage across a capacitor to the current density:

$$\frac{dV_{mem}}{dt} = -\frac{1}{C_{mem}}\,J_{mem} \tag{1}$$

The net current across the membrane, J_{mem}, was described by the sum of total transmembrane ion fluxes, ϕ_i^{tot} , for each ion, i:

$$J_{mem} = \sum_i F\,z_i\,\phi_i^{tot} \tag{2}$$

Where F is Faraday's constant ($96,485\,C/mol$) and z_i is the charge of the ion.

In turn, the total transmembrane flux of an ion could have contributions from possible ion pump/transporter activity (ϕ_i^{pump}), passive base transmembrane flux (ϕ_i^{mem}), passive transmembrane flux from open ion channel presence (ϕ_i^{chan}), or passive transport between GJ-coupled cells (ϕ_i^{gj}):

$$\phi_i^{tot} = \phi_i^{pump} + \phi_i^{mem} + \phi_i^{chan} + \phi_i^{gj} \tag{3}$$

Passive base transmembrane flux of an ion, i , was calculated using the GHK flux equation:

Ion	Intracellular (mM)	Extracellular (mM)	0.1x MMR (mM)
Na^{+}	21.3	101.8	10.0
K^{+}	91.0	3.7	0.5
Cl^{-}	40.2	37.6	9.0
Ca^{2+}	50e-6	1.5	0.1
$Prot^{-}$	47.0	10.0	0.0
ATP^{2-}	2.5	0.0	0.0
ADP^{-1}	0.1	0.0	0.0
Pi^{-1}	0.1	0.0	0.0
H^{+}	4e-5	4e-5	4e-5
HCO_3^{-}	10.0	10.0	1.0
M^{-}	15.1	47.9	1.5

Supplementary Table 1: Initial simulation concentrations in the intracellular space (cytosol), extracellular space (intercellular regions), and global environmental space surrounding the cell cluster. All bioelectrical variables, such as V_{mem}, V_{env} , all net charge, and ion currents were zero at time zero, and all voltage-sensitive gap junctions were 100% open at time zero.

$$\phi_i^{mem} = \frac{z_i\, V_{mem}\, F\, P_i^{mem}}{R\, T} \left(\frac{c_i^{cell} - c_i^{env}\, exp\left(-\frac{z_i V_{mem} F}{RT}\right)}{1 - exp\left(-\frac{z_i V_{mem} F}{RT}\right)} \right) \tag{4}$$

Where P_i^{mem} is the base permeability of the ion through the membrane, $R = 8.3145\, J/mol\, K$ is the ideal gas constant, $T = 310\, K$ is the temperature, c_i^{cell} and c_i^{env} are the concentrations of the ion inside and outside of the cell, respectively. Initial values of c_i^{cell} and c_i^{env}, as well as base membrane permeabilities (i.e. in the absence of specific channel activity, are listed in Table 1).

Gap junctions are channels that bridge the cytoplasm of two cells, enabling the passage of chemical and electrical signals. Ion flux through GJ was modeled using the Nernst-Planck equation:

$$\phi_i^{gj} = -\beta_{gj}\, D_i^{gj}\, \nabla c_{gj} - \frac{\beta_{gj}\, D_i^{gj}\, z_i\, q}{k_b T}\, c_i\, \nabla V_{gj} \tag{5}$$

Where β_{gj} is the voltage-sensitivity scaling parameter (modeled as described in [4]), D_i^{gj} is the effective diffusion constant of the ion through gap junctions, ∇c_{gj} is the concentration gradient of the ion across the gap junction, ∇V_{gj} is the difference in V_{mem} across the two gap junction coupled cells,$q = 1.6e^{-19}\, C$ is the unit charge constant, and k_b is Boltzmann's constant.

The total change in ion concentration in a single cell from all sources of transmembrane flux was calculated by taking the divergence of all total transmembrane flux for ion i:

$$\frac{\partial c_{cell}^{i}}{\partial t} = -\nabla \cdot \phi_{tot}^{i} \tag{6}$$

Basic Test Simulations

Basic test simulations were performed on a small circular cluster of cells (in 0.1X MMR – normal Xenopus medium used in all our experiments) to confirm that fundamental results were consistent with expectations (Supplementary Fig. 5). A sequence of forced, transient membrane permeability perturbations followed by a brief period of increased environmental K+ concentration was simulated, and the Goldman-Hodgkin-Katz (GHK) equation was

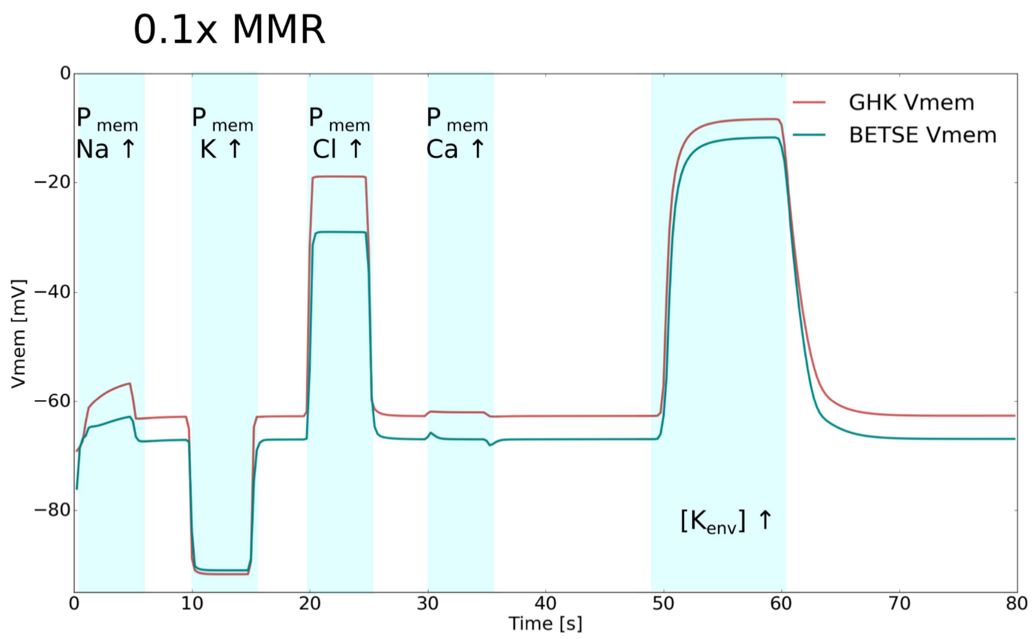

Supplementary Fig. 5: Test simulation confirming expected changes to V_{mem} with increases to membrane permeability to sodium, potassium, chloride, calcium, and increased extracellular concentration of K+ ions for a single cell simulation in media with intra and extracellular concentrations equivalent to those listed in Table 1 (applicable to apical membranes).

used as a cross-check for V_{mem} values obtained from the BETSE simulator. The test sequence shown in Supplementary Fig. 5 forced five second increases in membrane permeability (P_{mem}) of Na^+, K^+, Cl^-, and Ca^{2+}, where each membrane permeability value was increased from its starting state for five seconds before returning to its original value, and each perturbation was separated by an additional five seconds to allow the system to recover. The starting (i.e., non-perturbed) P_{mem} for Na^+, K^+, Cl^-, and Ca^{2+} were 6.7e^{-10}, 23.3e^{-10}, 2.0e^{-10}, and 2.0e^{-10} m/s, respectively, and perturbations transiently increased these values by a factor of ten. The test sequence was completed by increasing environmental K^+ concentrations to 35 mM for ten seconds before returning them to their original value (see Supplementary Table 1). Overall, all perturbations showed their expected V_{mem} deviations, and very good correspondence was seen between BETSE V_{mem} calculations and those estimated using the GHK equation (Supplementary Fig. 5). BETSE-calculated V_{mem} shows a small constant hyperpolarization offset that is attributable to the action of the electrogenic Na^+/K^+-ATPase pumps, which the base GHK equation does not take into account (Supplementary Fig. 5).

HCN2 Channel Model

A kinetic model of the voltage sensitive HCN2 channel was obtained from the work of Moosmang *et al.* [5]. The Moosman *et al.* voltage gated HCN2 model is a Hodgkin-Huxley style differential equation system, which defines m_{inf} and m_{tau} as two functions of V_{mem}; here we shift the $V_{1/2}$ value of the channel by +20 mV to account for the shift induced by a cyclic nucleotide such as cAMP [6–8]:

$$m_{inf} = \frac{1.0}{1 + exp(\frac{V+79.0}{6.2})} \tag{7}$$

$$m_{tau} = 184.0 \tag{8}$$

where the parameter m changes as a function of time according to:

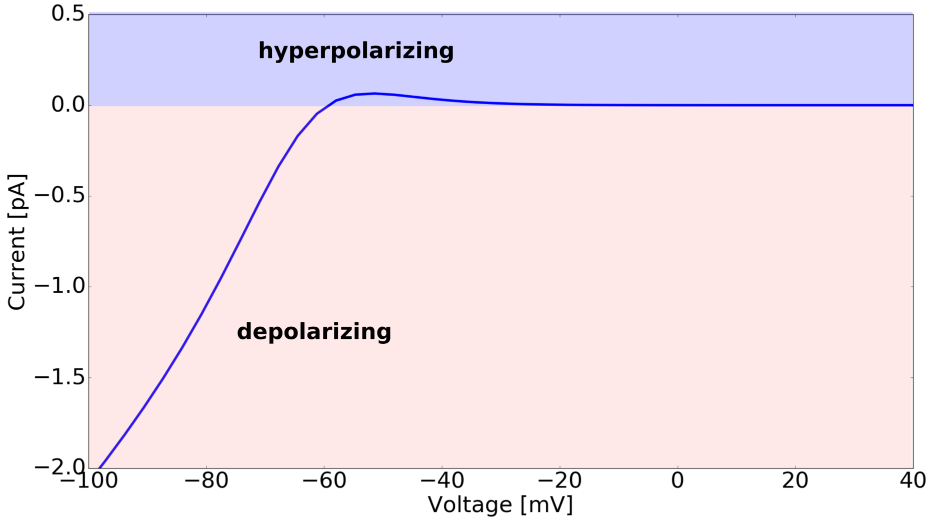

Supplementary Fig. 6: Simulated voltage clamp curve for the modeled HCN2 channel operating with intra and extracellular concentrations equivalent to those listed in Table 1 (applicable to channel behavior on apical membranes). Blue shaded region highlights the area where the channel current is predicted to be hyperpolarizing of V_{mem}, while the red shaded region highlights the area where the channel current is predicted to be depolarizing of V_{mem}.

$$\frac{\partial m}{\partial t} = \frac{m_{inf} - m}{m_{tau}} \tag{9}$$

In BETSE, the time- and V_{mem}-dependent parameter m was used to modulate the membrane permeability to K^+ ions according to:

$$P_K^{HCN} = P_K^{max}\, m \tag{10}$$

where $P_K^{max} = 6.7e^{-10}\ m/s$ was the maximum permeability for the HCN2 channel in wild type Xenopus neurula and $P_K^{max} = 6.7e^{-8}\ m/s$ was used for embryos overexpressing HCN2.

During each time-step, the membrane permeability would be updated by first using Eqs 9 to update m, then using 10 to determine the permeability change of the membrane. The HCN2 channel contribution to transmembrane K^+ flux was calculated using:

$$\phi_K^{HCN2} = \frac{z_K\, V_{mem}\, F\, P_K^{HCN}}{R\, T} \left(\frac{c_K^{cell} - c_K^{env}\, exp\left(-\frac{z_K V_{mem} F}{RT}\right)}{1 - exp\left(-\frac{z_K V_{mem} F}{RT}\right)} \right) \tag{11}$$

As the HCN channels are also permeable to Na^+ and Ca^{2+}, membrane permeabilities of $P_{Na}^{HCN2} = 0.2\, P_K^{HCN2}$ and $P_{Ca}^{HCN2} = 0.05\, P_K^{HCN}$ were calculated from P_{HCN}^K, and equations analogous to 11 were used to determine components of transmembrane flux for HCN-specific Na^+ and Ca^{2+} ions.

The simulated current-voltage curve for the modeled HCN2 channel is shown in Supplementary Fig. 6.

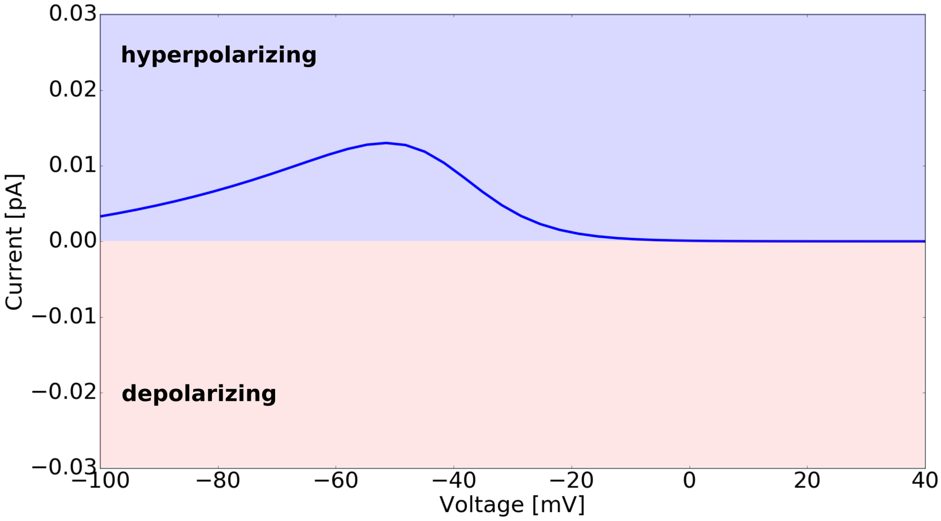

Supplementary Fig. 7: Simulated voltage clamp curve for the modeled Kir channel operating with intra and extracellular concentrations equivalent to those listed in Table 1 (applicable to channel behavior on apical membranes). Blue shaded region highlights the area where the channel current is predicted to be hyperpolarizing of V_{mem}, while the red shaded region highlights the area where the channel current is predicted to be depolarizing of V_{mem}.

Kir Channel Model

The Kir channel was modeled in terms of a voltage-sensitive cytosolic Mg^{2+} ion block of the channel [9, 10], using the following formula to describe channel activity:

$$\phi_K^{Kir2.1} = \frac{z_K\, V_{mem}\, F\, P_K^{Kir2.1}}{R\,T} \left(\frac{c_K^{cell} - c_K^{env}\, exp\left(-\frac{z_K V_{mem} F}{RT}\right)}{1 - exp\left(-\frac{z_K V_{mem} F}{RT}\right)} \right) \tag{12}$$

$$P_K^{Kir2.1} = P_K^{max} \left(\frac{1}{1 + \left(\frac{[Mg]}{K_{Mg}^{Kir2.1}}\right)} \right) \tag{13}$$

$$K_{Mg}^{Kir2.1} = K_{Mg}^{o}\, exp\left(\frac{-V_{mem}\, z_{Mg}^{eff}\, F}{R\,T} \right) \tag{14}$$

Here $P_K^{max} = 3.33e^{-9}\, m/s$, $[Mg] = 0.5\, mol/m^3$,$K_{Mg}^{o} = 0.025\, mol/m^3$, and $z_{Mg}^{eff} = 1.5$.

The simulated current-voltage curve for the modeled Kir2.1 channel is shown in Supplementary Fig. 7.

Nicotinic Acetylcholine Receptor Model

The nicotinic acetylcholine receptor was modeled assuming nicotine concentration activates the open state of a channel with permeability to Na^+ and K^+ ions with a stoichiometry of 1:1 [11, 12]. The maximum permeability of the nAChR was set as $P_{max}^{nAChR} = 1.33e^{-10}\, m/s$.

Modeling Effects of Nicotine on Ion Channels

While nicotine is well known to agonize the nicotinic acetylcholine receptor (nAChR) [13, 14], it can also directly block a variety of K^+ ion channels [15–18], including the HCN channels [19, 20]. Therefore, the agonizing effect of nicotine on nAChR, as well as the direct nicotine block of Kir and HCN2 channels was included in models using a standard Hill function to describe the influence of nicotine on the channel state as a function of nicotine concentration. To simulate nicotine treatment, nicotine was introduced from the environmental boundaries of the model at a concentration of 0.62 mM.

The HCN channel was modeled as directly blocked by nicotine with a saturation of the nicotine channel block to a level of 30%. The block of HCN by nicotine was modeled using the equation:

$$P^{HCN} = P^{HCN}_{max} \left(\frac{(1-0.7)}{1 + \left(\frac{cNicotine}{K_{nicotine}}\right)^{h_{nicotine}}} + 0.7 \right) \tag{15}$$

With $K_{nicotine} = 62\,nm$ and $h_{nicotine} = 7$ with a saturation at 30% block of channel state as described in experimental reports of nicotine related HCN channel block [19, 20].

The Kir channel was modeled as directly and fully blocked by nicotine. The block of Kir by nicotine was modeled using the equation:

$$P^{Kir} = P^{Kir}_{max} \left(\frac{1}{1 + \left(\frac{cNicotine}{K_{nicotine}}\right)} \right) \tag{16}$$

With $K_{nicotine} = 1.0\,mM$ for the direct block of nicotine by the Kir channel, as described in experimental reports of nicotine related Kir channel block [15].

The nAChR receptor was modeled as activated by nicotine with the function:

$$P^{Kir}_{K} = P^{nAChR}_{max} \left(\frac{\left(\frac{cNicotine}{K_{nicotine}}\right)}{1 + \left(\frac{cNicotine}{K_{nicotine}}\right)} \right) \tag{17}$$

With $K_{nicotine} = 83\,\mu M$ for the activation of the nAChR by nicotine, in accordance with the EC50 reported elsewhere [13].

Supplementary References

[1] Pietak A, Levin M. Exploring Instructive Physiological Signaling with the Bioelectric Tissue Simulation Engine. Front Bioeng Biotechnol. 2016;p. 55.

[2] Wright SH. Generation of Resting Membrane Potential. AJP: Advances in Physiology Education. 2004 Dec;28(4):139–142.

[3] Pietak A, Levin M. Bioelectric Gene and Reaction Networks: Computational Modelling of Genetic, Biochemical and Bioelectrical Dynamics in Pattern Regulation. J R Soc Interface. 2017 Sep;14(134).

[4] Harris AL, Spray DC, Bennett MV. Control of Intercellular Communication by Voltage Dependence of Gap Junctional Conductance. The Journal of Neuroscience. 1983;3(1):79–100.

[5] Moosmang S, Stieber J, Zong X, Biel M, Hofmann F, Ludwig A. Cellular Expression and Functional Characterization of Four Hyperpolarization-Activated Pacemaker Channels in Cardiac and Neuronal Tissues. European Journal of Biochemistry. 2001;268(6):1646–1652.

[6] Xu X, Vysotskaya ZV, Liu Q, Zhou L. Structural Basis for the cAMP-Dependent Gating in the Human HCN4 Channel. J Biol Chem. 2010 Nov;285(47):37082–37091.

[7] Lyashchenko AK, Redd KJ, Goldstein PA, Tibbs GR. cAMP Control of HCN2 Channel Mg2+ Block Reveals Loose Coupling between the Cyclic Nucleotide-Gating Ring and the Pore. PLOS ONE. 2014 Jul;9(7):e101236.

[8] Kusch J, Thon S, Schulz E, Biskup C, Nache V, Zimmer T, et al. How Subunits Cooperate in cAMP-Induced Activation of Homotetrameric HCN2 Channels. Nature Chemical Biology. 2011 Dec;8(2):162–169.

[9] Horie M, Irisawa H, Noma A. Voltage-Dependent Magnesium Block of Adenosine-Triphosphate-Sensitive Potassium Channel in Guinea-Pig Ventricular Cells. J Physiol. 1987 Jun;387:251–272.

[10] Makary SM, Claydon TW, Enkvetchakul D, Nichols CG, Boyett MR. A Difference in Inward Rectification and Polyamine Block and Permeation between the Kir2.1 and Kir3.1/Kir3.4 K+ Channels. J Physiol. 2005 Nov;568(Pt 3):749–766.

[11] Albuquerque EX, Alkondon M, Pereira EFR, Castro NG, Schrattenholz A, Barbosa CTF, et al. Properties of Neuronal Nicotinic Acetylcholine Receptors: Pharmacological Characterization and Modulation of Synaptic Function ,. J Pharmacol Exp Ther. 1997 Mar;280(3):1117–1136.

[12] Torrão AS, Britto LRG. Neurotransmitter Regulation of Neural Development: Acetylcholine and Nicotinic Receptors. Anais da Academia Brasileira de Ciências. 2002 Sep;74(3):453–461.

[13] Briggs CA, McKenna DG, Piattoni-Kaplan M. Human Alpha 7 Nicotinic Acetylcholine Receptor Responses to Novel Ligands. Neuropharmacology. 1995 Jun;34(6):583–590.

[14] Papke RL, Dwoskin LP, Crooks PA. The Pharmacological Activity of Nicotine and Nornicotine on nAChRs Subtypes: Relevance to Nicotine Dependence and Drug Discovery. J Neurochem. 2007 Apr;101(1):160–167.

[15] Hanna ST, Cao K, Sun X, Wang R. Mediation of the Effect of Nicotine on Kir6.1 Channels by Superoxide Anion Production. J Cardiovasc Pharmacol. 2005 May;45(5):447–455.

[16] Wang H, Yang B, Zhang L, Xu D, Wang Z. Direct Block of Inward Rectifier Potassium Channels by Nicotine. Toxicol Appl Pharmacol. 2000 Apr;164(1):97–101.

[17] Wang H, Shi H, Wang Z. Nicotine Depresses the Functions of Multiple Cardiac Potassium Channels. Life Sci. 1999;65(12):PL143–149.

[18] Wang H, Shi H, Zhang L, Pourrier M, Yang B, Nattel S, et al. Nicotine Is a Potent Blocker of the Cardiac A-Type K(+) Channels. Effects on Cloned Kv4.3 Channels and Native Transient Outward Current. Circulation. 2000 Sep;102(10):1165–1171.

[19] Griguoli M, Maul A, Nguyen C, Giorgetti A, Carloni P, Cherubini E. Nicotine Blocks the Hyperpolarization-Activated Current Ih and Severely Impairs the Oscillatory Behavior of Oriens-Lacunosum Moleculare Interneurons. J Neurosci. 2010 Aug;30(32):10773–10783.

[20] Kodirov SA, Wehrmeister M, Colom LV. Modulation of HCN Channels in Lateral Septum by Nicotine. Neuropharmacology. 2014 Jun;81:274–282.
